# ﻿Integrative taxonomy of the leaf-beetle genus *Gonioctena* Chevrolat, 1836 in Taiwan (Coleoptera, Chrysomelidae, Chrysomelinae, Gonioctenini) reveals new synonymies and one new species

**DOI:** 10.3897/zookeys.1120.87526

**Published:** 2022-09-05

**Authors:** Chi-Feng Lee, Chia-Hung Hsieh

**Affiliations:** 1 Applied Zoology Division, Taiwan Agricultural Research Institute, Taichung 413, Taiwan Taiwan Agricultural Research Institute Taichung Taiwan; 2 Department of Forestry and Nature Conservation, Chinese Culture University, Taipei 111, Taiwan Chinese Culture University Taipei Taiwan

**Keywords:** New host plants, new species, new synonym, nomenclature, taxonomy

## Abstract

Taiwanese species of *Gonioctena* are revised based on morphological, molecular, and ecological information. *Gonioctenasubgeminata* (Chen, 1934), *G.tredecimmaculata* (Jacoby, 1888), *G.kamikawai* (Chûjô, 1958), and *G.osawai* Kimoto, 1996 are redescribed. The study confirms that Phytodectatredecimmaculatusvar.taiwanensis Achard, 1924 should be considered a junior synonym of *G.tredecimmaculata* (Jacoby, 1888), along with two new junior synonyms, *G.ohmomoi*Cho et al. 2016 (**syn. nov.**) and *G.riyuetanensis*Cho et al. 2016 (**syn. nov.**). Phytodecta (Asiphytodecta) issikii Chûjô, 1958 (**syn. nov.**) is treated as a junior synonym of *Gonioctenascutellaris* Baly, 1862, which is removed from synonym with *G.fulva* (Motschulsky, 1861) (**stat. rev.**) and redescribed. *Gonioctenathoracica* Baly, 1862 (**syn. nov.**), *G.dichroa* Fairmaire, 1888 (**syn. nov.**), and *G.foochowensis* Gruev, 1989 (**syn. nov.**) are proposed as junior synonyms of *G.scutellaris* Baly, 1862. A new species, *G.liui***sp. nov.**, is described and differentiated ecologically from *G.scutellaris*. *Gonioctenanigroplagiata* Baly, 1862 is newly recorded from Matsu Islands and redescribed. Host plants and biological information are provided for all Taiwanese species. Lectotypes are designated for *G.scutellaris* Baly, 1862, *G.thoracica* Baly, 1862, and *G.nigroplagata* Baly, 1862.

## ﻿Introduction

The genus *Gonioctena* Chevrolat, 1836 is the largest chrysomeline genus, with 94 species in the Oriental, Palaearctic, and Nearctic regions (Brown 1942; Cho and Borowiec 2016). Five species of *Gonioctena* have been recorded from Taiwan previously: G.tredecimmaculatusvar.taiwanensis, described by Achard (1924); *G.issikii* and *G.kamikawai*, described by Chûjô (1958), *G.subgeminata* (Chen, 1934) also recorded by Chûjô in the same paper; and *G.osawai*, described by Kimoto (1996).

Taxonomic problems with some Taiwanese species are complex. Chûjô (1958) treated G.tredecimmaculatavar.taiwanensis (Archard, 1924) as an infraspecific variation of *G.tredecimmaculata* (Jacoby, 1888), which was also used by Bezděk (2002). However, Cho et al. (2016) regarded G.tredecimmaculatavar.taiwanensis as a distinct species from *G.tredecimmaculata* and described two new species, *G.ohmomoi* and *G.riyuetanensis*, both similar to *G.tredecimmaculata* with subtle differences in aedeagal structure and color patterns of the dorsum. A better solution was sought using molecular phylogenetics and biological information, the results of which are presented here.

Some *Gonioctena* species are extremely variable in coloration but similar in external morphology, such as species in Finland (Silfverberg 1994), Japan (Takizawa 2007), and Korea (Cho and Lee 2008). Species identity depends heavily on male aedeagi (Takizawa 2007; Cho and Lee 2008). However, taxonomic problems occur when sympatric populations and their aedeagi are less diagnostic, such as how Chinese and Taiwanese populations of *Gonioctenatredecimmaculata* were treated by Cho et al. (2016). These problems cannot be solved only on the characters of the male aedeagi, and so molecular studies and niche separation must be considered.

The Taiwan Chrysomelid Research Team (TCRT; website) was founded by Chi-Feng Lee in 2005 to inventory of chrysomelid species in Taiwan. Today, the team is comprised of 10 amateurs, led by Chi-Feng Lee, and our specimen and biological data are stored at the TARI. As part of this volunteer-led inventory, we observed host plants for all species of *Gonioctena* in the field and were able to rear females and larvae into the laboratory for many years. We present our results related to *Gonioctena*, including the two previously unrecorded species and new biological information are presented here.

## ﻿Materials and methods

The study is based on 658 individuals of eight species of Gonioctena, from museum collections and our rearing.

### ﻿Morphological study and rearing

For rearing studies, larvae were placed in small glass containers (diameter 142 mm × height 50 mm) with cuttings from their host plants. When mature larvae began searching for pupation sites, they were transferred to smaller plastic containers (diameter 90 mm × height 57 mm) filled with moist soil (~ 80% of container volume).

For taxonomic study, the abdomens of adults were separated from the forebodies and boiled in 10% KOH solution, followed by washing in distilled water to prepare genitalia for illustrations. The genitalia were then dissected from the abdomens, mounted on slides in glycerin, and studied and drawn using a Leica M165 stereomicroscope. For detailed examinations a Nikon ECLIPSE 50i microscope was used.

At least three pairs from each species were examined to delimit variability of diagnostic characters. For species collected from more than one locality, at least one pair from each locality was examined. Length was measured from the anterior margin of the eye to the elytral apex, and width at the greatest width of the elytra.

Specimens studied or mentioned herein are deposited at the following institutes and collections:

**DBET** Department of Biodiversity and Evolutionary Taxonomy, University of Wrocław, Wrocław, Poland;

**KMNH**Kitakyushu Museum of Natural History and Human History, Kitakyushu, Japan [Yûsuke Minoshima];

**MCZC**Museum of Comparative Zoology, Harvard University, Massachusetts, USA;

**MNHUB**Museum für Naturkunde, Leibniz-Institut für Evolutions- und Biodiversitätsforschung an der Humboldt-Universität zu Berlin, Berlin, Germany;

**MNHN**Museum national d’Histoire naturelle, Paris, France [Antoine Mantilleri, Christophe Rivier];

**NHMUK**The Natural History Museum, London, UK [Michael F. Geiser, Maxwell V. L. Barclay];

**NMPC**National Museum, Praha, Czech Republic;

**SEHU**Laboratory for Systematic Entomology, Hokkaido University, Sapporo, Japan;

**TARI**Taiwan Agricultural Research Institute, Taichung, Taiwan;

**ZMMU**Zoological Museum, Moscow State University, Moscow, Russia [Vladimir Savitsky].

Exact label data are cited for all type specimens of described species; a double slash (//) divides the data on different labels and a single slash (/) divides the data in different rows. Other comments and remarks are in square brackets: [p] – preceding data are printed, [h] – preceding data are handwritten, [w] – white label, [y] – yellow label, [g] – green label, [b] – blue label, and [r] – red label.

### ﻿Molecular study

#### DNA extraction, PCR amplification, and DNA sequencing

Genomic DNA extractions were conducted using Tissue Genomic DNA Extraction Mini Kit (Abundance Life Science, Kaohsiung, Taiwan). The partial mitochondrial cytochrome c oxidase subunit I (COI) gene sequence (ca. 700 bp) was amplified by PCR with the universal primer set, LCO1490 (5’-GGTCAACAAATCATAAAGATATTGG-3’) and HCO2198 (5’-TAAACTTCAGGGTGACCAAAAAATCA-3’) (Folmer et al. 1994). PCR amplifications were conducted using 1 μl of each template DNA in a total reaction volume of 25 μl containing 0.15 μM of dNTPs, 2.5 mM of MgCl, 0.75 units of Taq DNA polymerase, and 0.6 μM of each primer in a Veriti thermal cycler (Applied Biosystems, Foster City, CA, USA). The PCR ampliﬁcation was an initial denaturation for 5 min at 95 °C, then 35 cycles of denaturation for 30 sec at 95 °C, annealing for 30 sec at 52 °C, elongation for 45 sec at 72 °C, and a ﬁnal extension at 72 °C for10 min. The PCR products were separated in 1.5% agarose gel using electrophoresis, stained with an FluoroDye (Smobio, Hsinchu, Taiwan). PCR products were purified using a PCR Clean Up System (Viogene, Taipei, Taiwan) and sequenced on an ABI 3730 DNA Analyzer (Applied Biosystems, CA, USA) using an ABI PRISM Terminator Cycle Sequencing Ready Reaction Kit, v. 3.1 (Applied Biosystems, CA, USA), and sequencing reactions were carried out by the Genomics Company, New Taipei City, Taiwan.

#### Sequence analyses and phylogenetic reconstruction

COI sequences were assembled using Seqman II software (Lasergene, Madison, WI, USA). Multiple sequence alignments were constructed using the default settings in Muscle in MEGA v. 7.0 (Tamura et al. 2021). The best nucleotide substitution model (T92+G+I) for phylogenetic analysis was estimated using the ModelTest in MEGA v. 7.0). Phylogenetic trees were reconstructed using maximum likelihood (**ML**) with 1000 bootstrap replications for nodal supports in MEGA v. 7.0. Bayesian inference (**BI**) was also used to reconstruct the phylogenetic tree using MrBayes v. 3.2 (Ronquist et al. 2012). Two runs of four independent Metropolis-coupled Markov chain Monte Carlo (MCMC) analyses were run for 1 × 10^6^ generations and sampled every 1000 generations with a burn-in length of the initial 10% generations. The genetic distance was calculated based on the Kimura 2-parameter model using MEGA v. 7.0.

## ﻿Results

**Figure 1.**
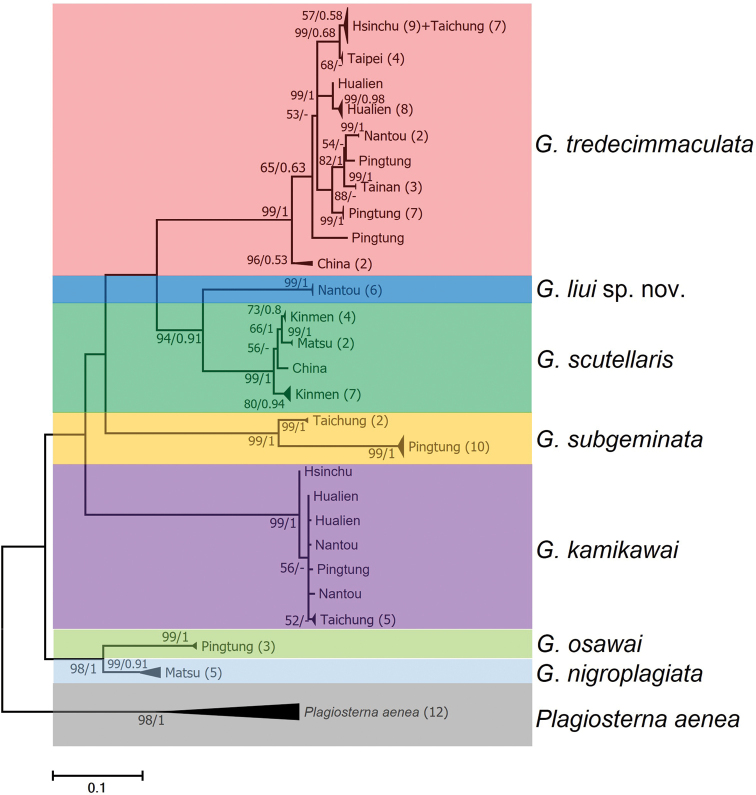
Maximum-likelihood phylogeny of *Gonioctena* based on mitochondrial COI sequences. Numbers at the nodes are maximum-likelihood bootstrap values and Bayesian posterior probabilities.

In total, 110 COI sequences (558 bp) from 98 leaf beetle specimens representing all seven Taiwan species of *Gonioctena* and 12 specimens of the outgroup, *Plagiosternaaenea* Linnaeus, 1758, were used to reconstruct a phylogeny. All sequences have been submitted to GenBank (Suppl. material 1). Maximum likelihood tree topology and bootstrap approach were less to support a false phylogenetic hypothesis (Douady et al. 2003). Maximum likelihood tree was the better topology to show in this study. Molecular phylogenetic analysis revealed that each *Gonioctena* species was divided into a distinct clade with high nodal support values (Fig. 1). Molecular data indicated an unambiguous linkage of *G.liui* sp. nov. and supported a close relationship with *G.scutellaris*. Morphological variations of *G.tredecimmmaculata* observed between China and Taiwan were revealed to represent distinct clades in the molecular analysis and are designated as the China clade and Taiwan clade. Interspecies genetic distances of *Gonioctena* were between 0.102 and 0.220 (Table 1). The minimum and maximum interspecific genetic distance between *G.liui* sp. nov and other *Gonioctena* spp. were 0.107 and 0.193, respectively, sufficient to justify species level. The intraspecies genetic distances of *Gonioctena* were between 0.001 and 0.035 (Table 1). Low intraspecies genetic distances (0.001) were observed within *G.liui* sp. nov. samples (Table 1). Genetic distances between the China clade and Taiwan clade of *G.tredecimmmaculata* was 0.058.

**Table 1.** Genetic distances between and within species of *Gonioctena* from Taiwan based on mitochondrial COI region.

	Taxon	1	2	3	4	5	6	7	within
1	*G.tredecimmmaculata*								0.035
2	*G.liui* sp. nov.	0.154							0.001
3	*G.scutellaris*	0.153	0.107						0.016
4	*G.subgeminata*	0.204	0.193	0.182					0.039
5	*G.kamikawai*	0.186	0.175	0.171	0.220				0.008
6	*G.osawai*	0.186	0.167	0.175	0.203	0.198			0.007
7	*G.nigroplagiata*	0.166	0.148	0.164	0.197	0.168	0.102		0.021
8	*Plagiosternaaenea*	0.214	0.209	0.218	0.248	0.232	0.208	0.208	0.073

### ﻿Taxonomic account

#### ﻿ Gonioctena (Asiphytodecta) subgeminata

Taxon classificationAnimaliaColeopteraChrysomelidae

(Chen, 1934)

5C1DEB19-7D45-5BAB-9CE8-CF89033551FC

Figs 2A–C , 3 , 4

Phytodecta subgeminatus Chen, 1934: 75 (China: Guandong, Guanzhou).
Phytodecta (Asiphytodecta) subgeminatus : Chen 1935: 131 (catalogue); Chen 1936: 88 (catalogue); Chûjô 1958: 67 (Taiwan).
Asiphytodecta subgeminatus : Chen and Young 1941: 208 (key).
Gonioctena (Asiphytodecta) subgeminata : Gressitt and Kimoto 1963: 365 (China: Guandong); Kippenberg 2010: 432 (catalogue); Yang et al. 2015: 54 (China: Hunan, Zhejiang); Cho 2021 (China: Anhui, Jiangxi, Sichuan).
Gonioctena (Asiphytodecta) subgeminatus : Kimoto and Chu 1996: 52 (catalogue); Kimoto and Takizawa 1997: 369 (catalogue).
Gonioctena subgeminata : Takizawa et al. 1995: 7 (additional records in Taiwan)

##### Types.

Two syntypes should be deposited at the MNHUB but appear to be lost (Cho 2021).

##### Other material (n = 60).

**China**. Fujian: 1♂ (TARI), 建陽 (Jianyang), 黃坑 (Huangkeng), 場头 (Changtou), 700–950m, 23.VI.1960, leg. 姜勝巧 (S.-Q. Jiang); 1♀ (TARI), 建陽 (Jianyang), 黃坑 (Huangkeng), 桂林 (Guiling), 290m, 21.VI.1960, leg. 姜勝巧 (S.-Q. Jiang); **Taiwan**. Nantou: 1♀ (TARI), Baibara (= Meiyuan, 眉原), 24.III.1943, leg. A. Aoki; 1♂ (TARI), Horisha (= Puli, 埔里), 10.V.1913, leg. M. Maki; 1♂ (TARI), same locality, 10.IV.1919, leg. H. Kawamura (collector was not present on the card); 1♂ (TARI), Wanfengtsun (萬豐村), 2.IV.2008, leg. W.-T. Liu; 1♂ (TARI), same but with “18.IV.2011”; Pingtung: 1♂ (TARI), Shinsuiyei (= Chinshuiying, 浸水營), 17.III.1926, leg. S. Issiki; 1♀ (TARI), Tahanshan (大漢山), 5.IV.2009, leg. C.-F. Lee; 2♂, 1♀ (TARI), same but with “26.III.2013”; 12♂, 2♀ (TARI), same but with “18.IV.2018”; 3♂, 1♀ (TARI), same locality, 3.IV.2012, leg. Y.-T. Chung; 6♂, 2♀ (TARI), same but with “16.IV.2013”; 1♂, 1♀ (TARI), same but with “10.V.2013”; 2♂ (TARI), same but with “28.III.2016”; 2♂ (TARI), same but with “10.IV.2017”; 3♂ (TARI), same but with “17.IV.2017”; 1♂ (TARI), same but with “5.IV.2018”; 2♂ (TARI), same but with “9.IV.2018”; 1♂ (TARI), same but with “23.IV.2018”; 2♂ (TARI), same but with “27.IV.2020”; 1♂ (TARI), same but with“20.III.2021”; 1♂ (TARI), same but with “10.IV.2021”; 2♂ (TARI), same but with “15.IV.2021”; 2♂, 1♀ (TARI), same locality, 29.IV.2014, leg. J.-C. Chen.

##### Redescription.

**Figure 2.**
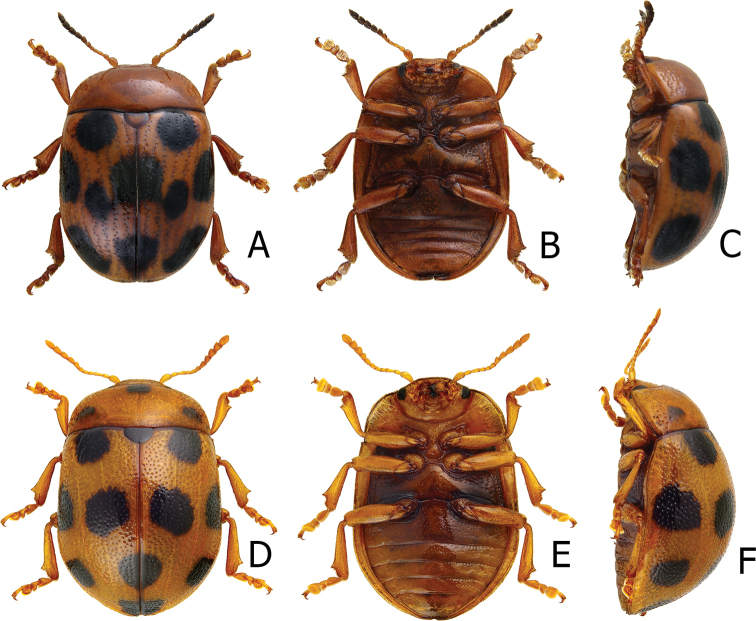
Habitus of Gonioctena (Asiphytodecta) subgeminata (Chen) and *G.* (*A.*) *tredecimmaculata* (Jacoby). **A**G. (A.) subgeminata, dorsal view **B** ditto, ventral view **C** ditto, lateral view **D**G. (A.) tredecimmaculata, dorsal view **E** ditto, ventral view **F** ditto, lateral view.

**Figure 3.**
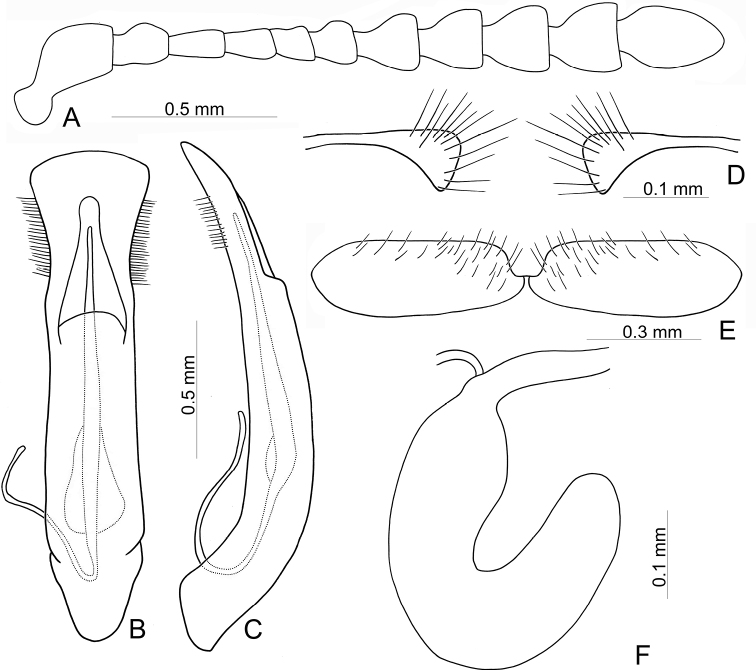
Diagnostic characters of Gonioctena (Asiphytodecta) subgeminata (Chen) **A** antenna **B** aedeagus, dorsal view **C** aedeagus, lateral view **D** gonocoxae **E** abdominal ventrite VIII **F** spermatheca.

**Adult** Length 5.2–6.3 mm, width 3.8–4.6 mm. Body color (Fig. 2A–C) yellowish brown, antennomeres VII–XI black, elytra with eleven black spots: three large spots along suture, anterior spot at basal 1/3, median at apical 1/3, the other at apices; one pair of large spots near base between suture and humeral calli; two pairs of spots at basal 1/3, large spot near lateral margin, small spot between large spot and one on suture; one pair of large spots at apical 1/3 near lateral margins. Antennae (Fig. 3A) with antennomere III–V slender, VI slightly swollen, VII–X strongly swollen, XI elongate oval, length ratios of antennomeres I–XI 1.0: 0.5: 0.5: 0.4: 0.3: 0.4: 0.5: 0.5: 0.5: 0.6: 0.9, length to width ratios of antennomeres I–XI 2.1: 1.3: 2.0: 1.7: 1.2: 1.0: 1.1: 1.1: 1.0: 1.0: 1.8. Pronotum 2.4× wider than long; lateral margins widest at base, convergent and strongly narrowed anteriorly; anterior angles strongly produced; anterior and lateral margins bordered, lateral margins barely visible in dorsal view; trichobothria absent on both anterior and posterior angles; disc covered with sparse, tiny punctures, both sides covered with much larger, denser punctures. Scutellum distinctly wider than long, narrowed posteriorly. Elytra 1.1× longer than wide; lateral margins slightly wider posteriorly, widest near middle; humeral calli well developed; disc covered with irregular punctures arranged in single rows; interspaces covered with fine, sparse punctures. Hind wing well developed. Aedeagus (Fig. 3B, C) with apical process widely rounded in dorsal view, lateral margins moderately narrowed in apical 1/4, with dense setae along lateral margins from near apex to apical 1/3; moderately curved in lateral view; endophallic sclerite extremely elongate, medially wider, basally membranous. Gonocoxae (Fig. 3D) slender, but apices wider and angular, disc with dense long setae. Ventrite VIII (Fig. 3E) transverse, with several short setae along outer margin. Receptacle of spermatheca (Fig. 3F) slightly swollen, not separated from pump; pump short and curved; sclerotized proximal spermathecal duct moderately long.

##### Diagnosis.

Adults of Gonioctena (Asiphytodecta) subgeminata are easily distinguished from those of other consubgeneric species, G. (A.) tredecimmaculata, by the following combination of the characters: lacking black spots on the pronotum (Fig. 2A) (with three black spots on the pronotum in G. (A.) tredecimmaculata (Fig. 2D)), yellowish brown scutellum (Fig. 2A) (black scutellum in G. (A.) tredecimmaculata (Fig. 2D)), three black spots on suture of elytra, at basal 1/3, median of apical 1/3, and at apices (Fig. 2A) (two black spots on suture of elytra, apical 1/3 and near apices in G. (A.) tredecimmaculata (Fig. 2D)), punctures on elytra arranged into longitudinal striae (Fig. 2A) (punctures on elytra entirely confused in G. (A.) tredecimmaculata (Fig. 2D)), apical process of aedeagus apically wider and apical margin widely rounded (Fig. 3B) (apically process of aedeagus apically narrow and apical margin narrowly rounded in G. (A.) tredecimmaculata (Figs 5B, 6B), slender gonocoxae with angular apices (Fig. 3D) (wide gonocoxae with irregular apical margin in G. (A.) tredecimmaculata (Figs 5D, 6D)), sparse, short setae along outer margins of ventrites VIII (Fig. 3E) (dense, short setae along outer margins of ventrite VIII in G. (A.) tredecimmaculata (Figs 5E, 6E)), relatively wider spermatheca (Fig. 3F) (more slender spermatheca in G. (A.) tredecimmaculata (Figs 5F, 6F)).

##### Host plants.

Fabaceae: *Pueraria* sp. (Yang et al. 2015) and *Calleryareticulata* (Benth.) Schot (Lee et al. 2016; present study, see below).

##### Biology.

**Figure 4.**
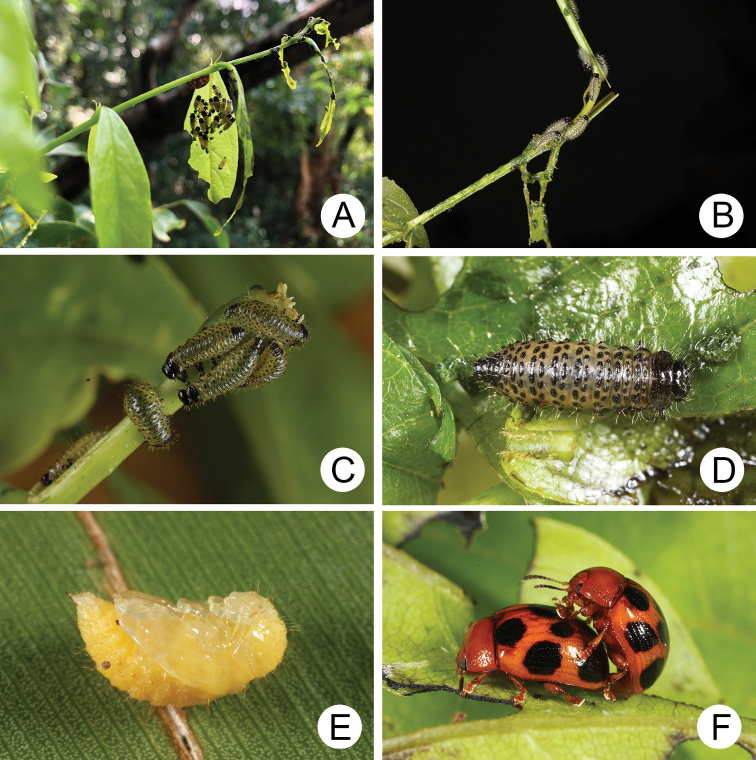
Natural history of Gonioctena (Asiphytodecta) subgeminata (Chen) in Taiwan **A** young larvae on the host plant, *Calleryareticulata* (Fabaceae) **B** second-instar larvae **C** third-instar larvae **D** fourth-instar larva **E** pupa **F** adult.

A large population of Gonioctena (Asiphytodecta) subgeminata was found in Tahanshan (大漢山), where Mr. Yi-Ting Chung (鍾奕霆) collected leaf beetles often. He collected early instar larvae feeding gregariously on young leaves (Fig. 4A–C) on 18 March 2022 from *Calleryareticulata*. In the lab, mature larvae (Fig. 4D) burrowed into the soil and built underground chambers for pupation on 22 March. Duration of the pupal stage (Fig. 4E) was seven days. Newly emerged adults (Fig. 4F) appeared in early April, but females failed to lay eggs. Thus, this species is likely univoltine since adults appear during spring (March to May).

##### Distribution.

China, Taiwan.

#### ﻿ Gonioctena (Asiphytodecta) tredecimmaculata

Taxon classificationAnimaliaColeopteraChrysomelidae

(Jacoby, 1888)

4F48034F-4466-5EE1-9EAD-81AEF20F0BAA

**Figure 5.**
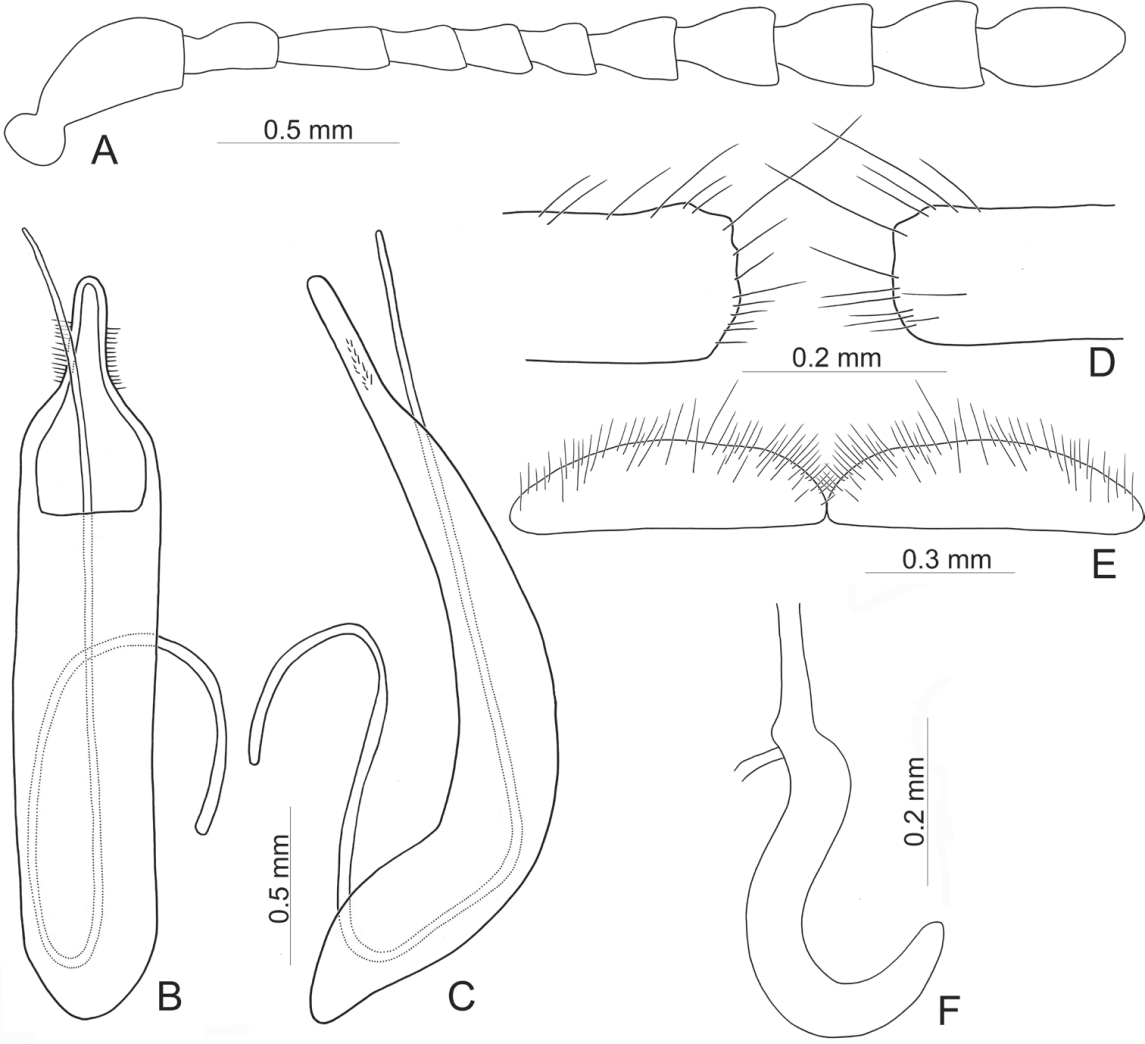
Diagnostic characters of Gonioctena (Asiphytodecta) tredecimmaculata (Jacoby) from Taiwan **A** antenna **B** aedeagus, dorsal view **C** aedeagus, lateral view **D** gonocoxae **E** abdominal ventrite VIII **F** spermatheca.

**Figure 6.**
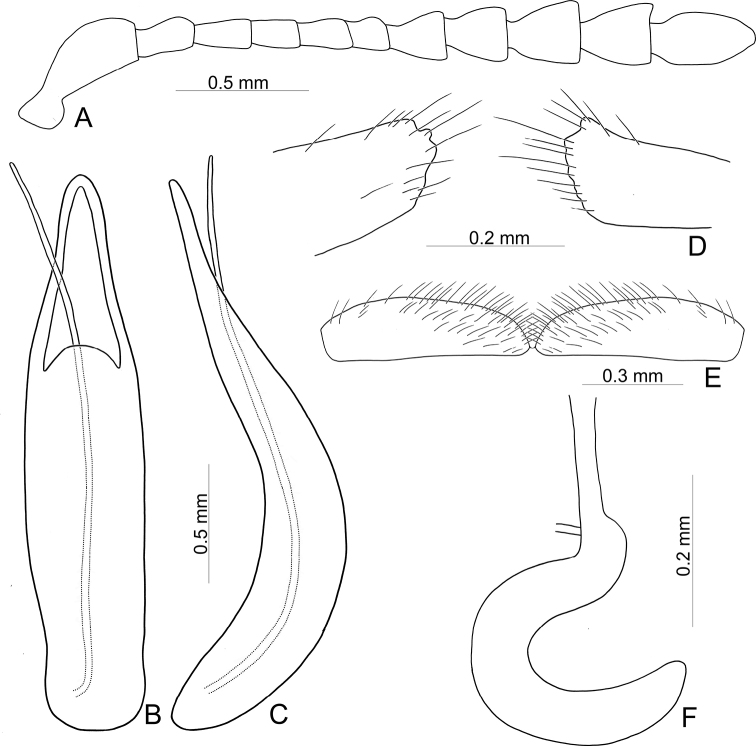
Diagnostic characters of Gonioctena (Asiphytodecta) tredecimmaculata (Jacoby) from China **A** antenna **B** aedeagus, dorsal view **C** aedeagus, lateral view **D** gonocoxae **E** abdominal ventrite VIII **F** spermatheca.

**Figure 7.**
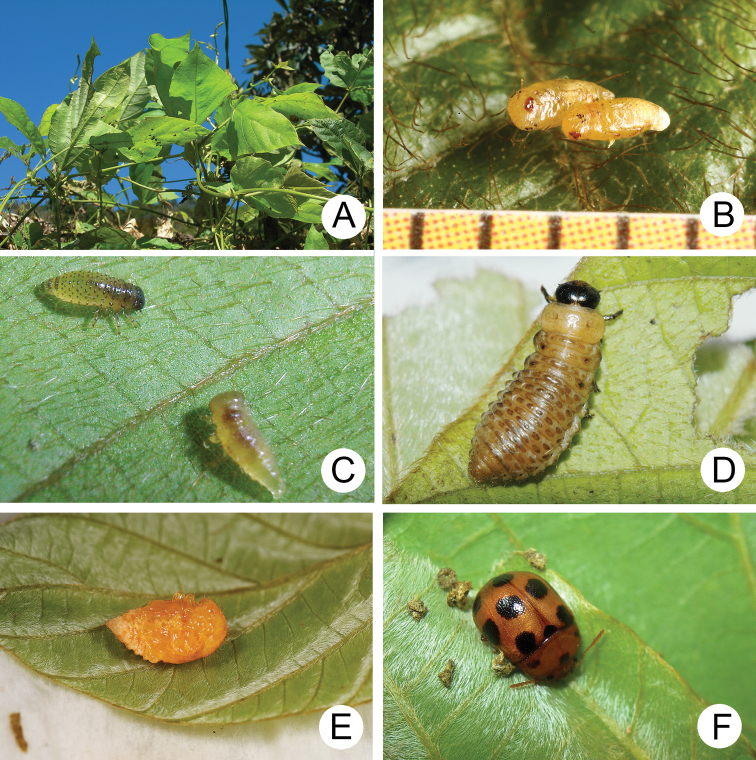
Natural history of Gonioctena (Asiphytodecta) tredecimmaculata (Jacoby) on host plant **A** host plant, Puerariamontanavar.montana (Fabaceae) **B** eggs **C** first-instar larvae **D** fourth-instar larva **E** pupa **F** adult.

Figs 2D–F , 5 , 6 , 7

Phytodecta tredecimmaculata Jacoby, 1888: 347 (China: Fujian); Jacoby 1890: 118 (diagnosis with P.nigrosparsus Fairmaire).
Phytodecta tredecimmaculatus : Winkler 1930: 1296 (catalogue); Chen 1934: 74 (part).
Phytodecta (s. str.) tredecimmaculatus : Weise 1916: 180 (catalogue).
Phytodecta (Asiphytodecta) tredecimmaculatus : Chen 1935: 132 (part); Chen 1936: 88 (part); Chûjô 1958: 64 (part).
Asiphytodecta tredecimmaculatus : Chen and Young 1941: 206 (key).
Gonioctena (Asiphytodecta) tredecimmaculata : Gressitt & Kimoto, 1963: 365 (part); Chûjô 1963: 386 (additional records in Taiwan); Kimoto 1966: 25 (one additional record in Taiwan); Kimoto 1967: 59 (China: Hong Kong); Kimoto 1969: 22 (additional records in Taiwan); Bezděk 2002: 9 (redefinition); Kippenberg 2010: 432 (catalogue); Yang et al. 2015: 54 (catalogue).
Gonioctena (Asiphytodecta) tredecimmaculatus : Kimoto and Chu 1996: 53 (catalogue); Kimoto and Takizawa 1997: 369 (catalogue).
Gonioctena tredecimmaculata : Kimoto 1989: 247 (additional records in Taiwan); Kimoto 1991: 8 (additional records in Taiwan).
Phytodecta tredecimmaculatus var. taiwanensis Achard, 1924: 34 (Taiwan); synonymized by Chûjô (1958). Synonym confirmed
Phytodecta (Asiphytodecta) tredecimmaculatus var. taiwanensis : Chen 1935: 132 (catalogue); Chen 1936: 88 (catalogue); Chûjô 1958: 65 (redescription).
Asiphytodecta tredecimmaculatus taiwanicus [sic!]: Chen and Young 1941: 207 (key).
Gonioctena (Asiphytodecta) tredecimmaculata taiwanica [sic!]: Gressitt & Kimoto, 1963: 359 (key)
Gonioctena (Asiphytodecta) taiwanensis : Cho et al. 2016: 363.
Gonioctena (Asiphytodecta) ohmomoi Cho et al. 2016: 365 (Taiwan: Hualien, Shoufeng (壽豐)). Syn. nov.
Gonioctena (Asiphytodecta) riyuetanensis Cho et al. 2016: 366 (Taiwan: Nantou, Sun Moon Lake (日月潭)) Syn. nov.

##### Types.

*Phytodectatredecimmaculata*: two syntypes (1♂ + 1♀) deposited at the MCZC were studied by Bezděk (2002) and Cho et al. (2016).

Phytodectatredecimmaculatusvar.taiwanensis: one syntype (♀) deposited at the NMPC was studied by Bezděk (2002) and Cho et al. (2016).

Gonioctena (Asiphytodecta) ohmomoi: The holotype (♂) and one paratype (♀) was deposited at the SEHU (Cho et al. 2016).

Gonioctena (Asiphytodecta) riyuetanensis: The holotype (♂) was deposited at the DBET (Cho et al. 2016).

##### Other material (n = 128).

**China**. Fujian: 1♀ (TARI), 崇安 (Chongan), 星村 (Xingcun), 掛墩 (Guadun), 950–1210m, 12.VI.1960, leg. 左丞 (C. Zuo), 1♀ (TARI), 建陽 (Jianyang), 黃坑 (Huangkeng), 大竹欄 (Dazhulan) – 先鋒嶺 (Xianfengling), 950–1170m, 2.V.1060, leg. 馬成林 (C.-L. Ma); **Taiwan**. Chiayi: 1♀ (TARI), Arisan (= Alishan, 阿里山), 17.V.1917, leg. T. Shiraki; 1♂ (TARI), Hsiting (隙頂), 1.VIII.2018, leg. Y.-T. Chung; Hsinchu: 2♀ (TARI), Shiigao (name of the tribe), Chikuto (= Maopu, 茅埔), 27–30.VI.1934, leg. M. Chûjô; 1♀ (TARI), Shinchiku (= Hsinchu, 新竹), 1–30.VII.1918, leg. J. Sonan, K. Miyake; 1♂ (TARI), Talu trail (大鹿林道), 22.X.2008, leg. H.-J. Chen; 1♂ (TARI), same locality, 22.IX.2012, leg. Y.-L. Lin; Hualien: 2♂, 2♀ (TARI), Chohsi (卓溪), 4.III.2016, leg. S.-P. Wu; 3♂, 2♀ (KMNH), Hungyeh Wenchuan (紅葉溫泉), 14.VI.1976, leg. H. Makihara; 1♀ (TARI), Mizuho (= Juisui, 瑞穗), 23.III.1935, leg. M. Chûjô; Ilan: 2♂, 3♀ (TARI), Ebosiyama (= Umutzushan, 烏帽子山), 17–21.V.1933, leg. M. Chûjô; 1♀ (TARI), Yingtzuling (鶯仔嶺), 15.IV.2012, leg. Y.-L. Lin; Kaohsiung: 1♀ (TARI), Chienshan (建山), 23.VI.2018, leg. B.-X. Guo; 1♂ (TARI), Hsiaokuanshan (小關山), 15.V.2016, leg. B.-X. Guo; 2♂ (KMNH), Liu Kui (六龜), 29.III.1986, leg. K. Baba; 2♂ (TARI), Paiyunshan (白雲山), 25.IV.2016, leg. U. Ong; 1♂ (TARI), Tengchih (藤枝), 4.VII.2011, leg. M.-H. Tsou; 1♀ (TARI), same locality, 18.IV.2013, leg. B.-X. Guo; 1♀ (TARI), same but with “3.VIII.2013”; 1♀ (TARI), same locality, 18.IV.2013, leg. Y.-T. Chung; Nantou: 1♂, 2♀ (TARI), Honbukei (= Penpuchi, 本部溪), 7.VII.1940, leg. M. Chûjô; 1♂ (TARI), Huisun Experimental Forest Station (惠蓀林場), 22.IV.2015, leg. B.-X. Guo; 1♀ (TARI), Lugu (鹿谷), 10.IX.2–14, leg. H.-T. Shih; 1♀ (KMNH), Hsitou (溪頭), 31.III.1980, leg. K. Sugiyama; 1♀ (TARI), Takeya (= Chienchityu, 乾溪仔), 8.VII.1940, leg. M. Chûjô; 1♂, 4♀ (TARI), Wanfengtsun (萬豐村), 2.IV.2008, leg. W.-T. Liu; 2♂ (TARI), same but with “9.VII.2008”; 1♀ (TARI), same but with “23.IV.2009”; 1♂ (TARI), same but with “13.IV.2010”; 2♂ (TARI), Wushe (霧社), 17.VIII.1984, leg. K. C. Chou; Pingtung: 1♂ (TARI), Koshun (= Hengchun, 恆春), 25.IV.-25.V.1918, leg. J. Sonan, K. Miyake, M. Yoshino; 1♂ (TARI), Nanjenshan (南仁山), 24.II.2009, leg. C.-F. Lee; 1♂ (TARI), Peihulushan (北湖呂山), 4.XI.2009, leg. M.-H. Tsou; 2♂, 3♀ (TARI), Peitawushan (北大武山), 22.IV.2013, leg. Y.-T. Chung; 1♂, 2♀ (TARI), same but with “8.V.2014”; 1♂ (TARI), same but with “2.V.2016”; 1♂ (TARI), same but with “10.X.2017”; 1♀ (TARI), Raisha (= Laiyi, 來義), 13.III.1926, leg. J. Sonan; 1♂ (TARI), Shouka (壽卡), 23.II.2013, leg. W.-C. Liao; 1♀ (TARI), Tahanshan (大漢山), 17.VII.2007, leg. M.-H. Tsou; 2♂ (TARI), same locality, 25.V.2008, leg. C.-F. Lee; 1♂ (TARI), same locality, 28.VIII.2010, leg. Y.-L. Lin; 1♂ (TARI), same locality, 26.VI.2012, leg. Y.-T. Chung; 1♂ (TARI), same but with “30.V.2014”; 2♀ (TARI), same but with “18.III.2016”; 1♂ (TARI), same but with “6.IX.2017”; 1♂ (TARI), same but with “28.VI.2020”; 1♂ (TARI), same but with “10.V.2021”; 4♂, 4♀ (TARI), Taiwu (泰武), 14.IX.2017, leg. Y.-T. Chung; 1♀ (TARI), Wanlite (萬里得), 1.III.2010, leg. M.-L. Cheng; Taichung: 1♂ (TARI), Hassenzan (= Pahsienshan, 八仙山), 4.VI.1942, leg. A. Mutuura; 1♂ (TARI), Kukuan (谷關), 14.IV.2019, leg. C.-C. Guo; 3♂ (TARI), same but with “28.IV.2019”; 1♂, 1♀ (TARI), Wushihkeng (烏石坑), 13.VII.2008, leg. M.-H. Tsou; 1♂ (TARI), same locality, 21.III.2013, leg. C.-F. Lee; Tainan: 4♀ (TARI), Kantoushan (崁頭山), 13.III.2010, leg. M.-H. Tsou; 5♂ (TARI), same but with “8.IV.2010”; 3♂, 3♀ (TARI), Meiling (梅嶺), 24.IV.2013, leg. B.-X. Guo; 1♂ (TARI), same but with “5.X.2015”; 2♀ (TARI), same locality, 2.XI.2015, leg. Y.-T. Chung; Taipei: 1♂ (TARI), Chungho (中和), 22.V.2010, leg. Y.-L. Lin; 1♂ (TARI), Fushan (福山), 11.IV.2021, leg. I.-H. Ku; 1♂ (TARI), Kotou (格頭), 27.III.2010, leg. H.-J. Chen; 1♂ (TARI), Urai (= Wulai, 烏來), 14.V.1933, leg. M. Chûjô; 1♀ (TARI), same locality, 16.IV.2008, leg. J.-F. Tsai; 1♀ (KMNH), Yangmingshan (陽明山), 28.IV.1970, leg. M. Yamamoto; Taitung: 1♀ (TARI), Anshuo (安朔), 27.VII.2011, leg. W.-T. Liu; 2♀ (TARI), Chipon (知本), 25.III.1935, leg. M. Chûjô; 1♂, 1♀ (TARI), same locality (= Tipon), 8.V.1943, leg. M. Chûjô; 1♂ (KMNH), Kueitien (歸田), 17.VI.1976, leg. H. Makihara; 1♂, 1♀ (TARI), Liyuan (栗園), 19.VI.2013, leg. C.-F. Lee; 1♂ (TARI), Motien (摩天), 18.VI.2013, leg. J.-C. Chen; 3♂, 1♀ (TARI), Taimali (太麻里), 20.III.2008, leg. P.-F. Wang; 1♀ (TARI), Taito (= Taitung, 台東), 25.II.-27.III.1919, leg. S. Inamura; Taoyuan: 1♂ (TARI), Hsuehwunao (雪霧鬧), 10.VI.2016, leg. Y.-L. Lin.

##### Redescription.

Length 6.9–8.4 mm, width 4.3–5.4 mm. Body color (Fig. 2D–F) yellowish brown, scutellum black, pronotum with three spots: one near apical margin at middle, and a pair of spots at sides; elytra with ten black spots, arranged as follows: two large spots along suture at apical 1/3 and near apices; one pair near base between suture and humeral calli; two pairs at basal 1/3, one large spot near lateral margin, another between it and suture; and one pair of large spots at apical 1/3 near lateral margins. Antennae (Fig. 5A) with antennomere III slender, IV–VI slightly swollen, VII moderately swollen, VIII–X strongly swollen, XI elongate oval, length ratios of antennomeres I–XI 1.0: 0.4: 0.5: 0.4: 0.3: 0.3: 0.4: 0.5: 0.5: 0.5: 0.7, length to width ratios of antennomeres I–XI 2.5: 1.9: 2.5: 1.6: 1.4: 1.3: 1.3: 1.3: 1.2: 1.2: 1.9. Pronotum 2.3× wider than long, lateral margins widest at base, convergent anteriorly, anterior angles strongly produced; anterior and lateral margins bordered, lateral margins barely visible in dorsal view; trichobothria absent on both anterior and posterior angles; disc covered with dense coarse punctures, interspaces with fine punctures, both sides covered with much larger, denser punctures. Scutellum distinctly wider than long, narrowed posteriorly. Elytra 1.1–1.2× longer than wide; lateral margins slightly wider posteriorly, widest near middle. Humeral calli well developed; disc covered with rather regular coarse punctures; interspaces covered with fine, sparse punctures. Hind wing well developed. Aedeagus (Fig. 5B, C) with apical process narrowly rounded in dorsal view, lateral margins slightly narrowed in apical 1/4, with dense short setae along lateral margins from near apex to apical 1/4; strongly curved at basal 1/3 in lateral view; endophallic sclerite extremely elongate. Gonocoxae (Fig. 5D) wide, but mesal margins subtruncate, with several long setae along apical and outer margins. Ventrite VIII (Fig. 5E) transverse, with dense long setae along outer margin. Receptacle of spermatheca (Fig. 5F) slender, not separated from pump; pump short and curved; sclerotized proximal spermathecal duct moderately long.

##### Variations.

Black spots on the dorsum are predominantly large in Chinese populations but extremely variable in Taiwanese populations. Diagnostic characters of Chinese populations (Fig. 6) are similar to those in Taiwan, but without dense, short setae along lateral margins of the aedeagus from near apex to apical 1/4 (Fig. 6B, C); and mesally narrowed apical margins of gonocoxae (Fig. 6D).

##### Diagnosis.

Gonioctena (Asiphytodecta) tredecimmaculata is easily distinguished from the other consubgeneric species, G. (A.) subgeminata by the following combination of the characters: three black spots on pronotum (Fig. 2D) (without black spots on pronotum in G. (A.) subgeminata (Fig. 2A)), black scutellum (Fig. 2D) (yellowish-brown scutellum in G. (A.) subgeminata (Fig. 2A)), two black spots on suture of elytra, one at apical 1/3 and the other near apices (Fig. 2D) (three black spots on suture of elytra, one at basal-median 1/3 median and apical 1/3, the other at apices in G. (A.) subgeminata (Fig. 2A)), punctures on elytra entirely confused (Fig. 2D) (punctures on elytra arranged into longitudinal striae in G. (A.) subgeminata (Fig. 2A)), apical process of aedeagus apically narrow and apical margin narrowly rounded (Figs 5B, 6B) (apical process of aedeagus apically wider and apical margin widely rounded in G. (A.) subgeminata (Fig. 3B)), wide gonocoxae with irregular apical margin (Figs 5D, 6D) (slender goncoxae with angular apices in G. (A.) subgeminata (Fig. 3D)), dense, short setae along outer margins of ventrite VIII (Figs 5E, 6E) (sparse short setae along outer margins of ventrite VIII in G. (A.) subgeminata (Fig. 3E)), more slender spermatheca (Figs 5F, 6F) (relatively wider spermatheca in G. (A.) subgeminata (Fig. 3F)).

##### Host plants.

Fabaceae: *Pueraria* sp. (Lee and Cheng 2007) and Puerariamontanavar.montana (Lour.) Merr. (Fig. 7A) (Frye et al. 2007; present study).

##### Biology.

Gonioctena (Asiphytodecta) tredecimmaculata populations are presumed to be univoltine and females are ovoviviparous. In Taiwan, overwintered adults appeared during early March. The females deposited larvae that were enclosed within the chorion (Fig. 7B), and they hatched after several hours (Fig. 7C). The larvae fed on leaves and the larval duration was eight days. Mature larvae (Fig. 7D) burrowed into soil and built underground chambers for pupation. Duration of the pupal stage (Fig. 7E) was 12 days. Newly emerged adults (Fig. 7F) appeared in mid-April. The biology of this species in In Jiangxi, China (Shen and Xue 1986) is similar to that of Taiwanese populations. However, overwintered adults appeared during late April. The larvae required ten to 12 days in May to mature and pupal duration was 13 to 16 days. The adults emerged during early June.

##### Remarks.

Genetic distance analyses showed that the genetic divergence between the China clade (*G.tredecimmaculata* (Jacoby, 1888)) and Taiwan clade (including G.tredecimmaculatusvar.taiwanensis Achard, 1924, *G.ohmomoi*Cho et al. 2016, and *G.riyuetanensis*Cho et al. 2016) of *G.tredecimmmaculata* has not reached to species level. In the field, adults of Taiwanese populations are found in lowlands and are monophagous on *Puerariamontana*. Thus, niche separation does not occur to these populations. The data support the hypothesis that they are members of the same species level lineage.

##### Distribution.

China, Taiwan.

#### ﻿ Gonioctena (Brachyphytodecta) scutellaris

Taxon classificationAnimaliaColeopteraChrysomelidae

Baly, 1862 stat. rev.

FB00E638-0FD8-58F1-95AB-27478DCFDD56

**Figure 8.**
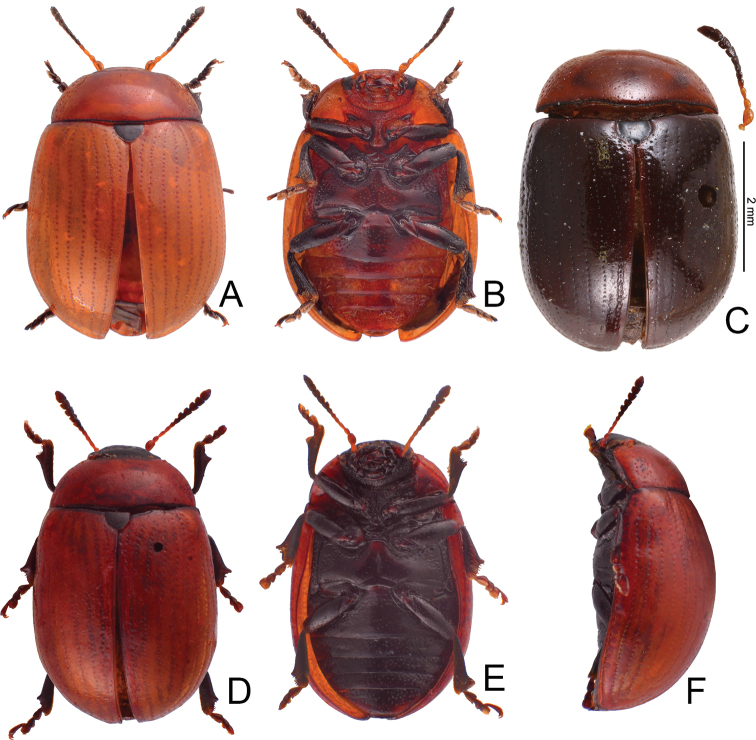
Habitus of Gonioctena (Brachyphytodecta) scutellaris Baly and G. (B.) fulva (Motschulsky) **A**G. (B.) scutellaris, typical form, dorsal view **B** ditto, ventral view **C** same species, color variation, syntype of *G.dichroa* Fairmaire **D**G. (B.) fulva, dorsal view **E** ditto, ventral view **F** ditto, lateral view.

**Figure 9.**
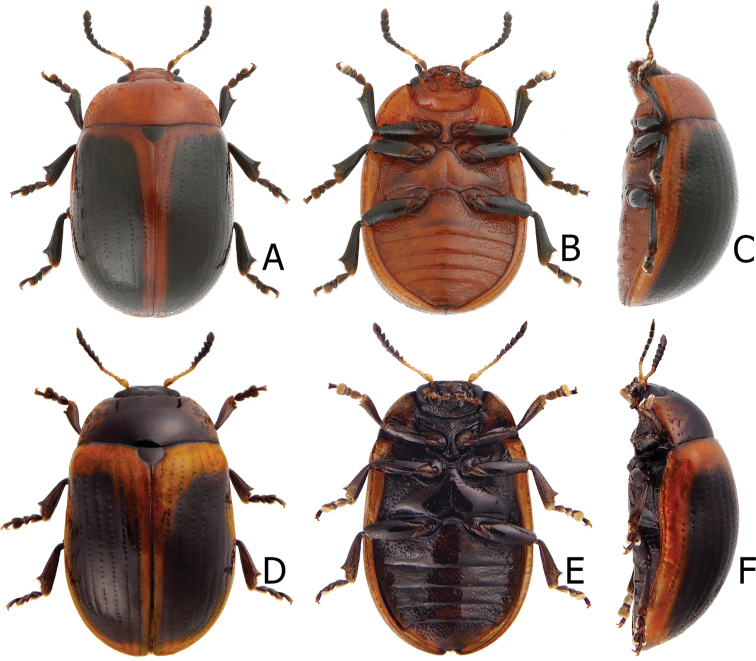
Habitus of Gonioctena (Brachyphytodecta) scutellaris Baly and G. (B.) liui sp. nov. **A**G. (B.) scutellaris, color variation, from Taiwan, dorsal view **B** ditto, ventral view **C** ditto, lateral view **D**G. (B.) liui sp. nov., dorsal view **E** ditto, ventral view **F** ditto, lateral view.

**Figure 10.**
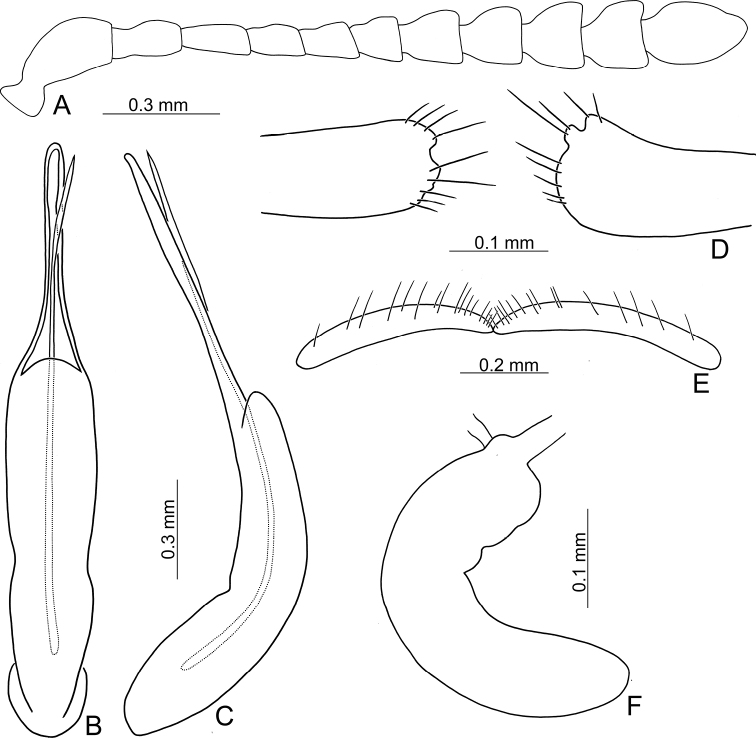
Diagnostic characters of Gonioctena (Brachyphytodecta) scutellaris Baly **A** antenna **B** aedeagus, dorsal view **C** aedeagus, lateral view **D** gonocoxae **E** abdominal ventrite VIII **F** spermatheca.

**Figure 11.**
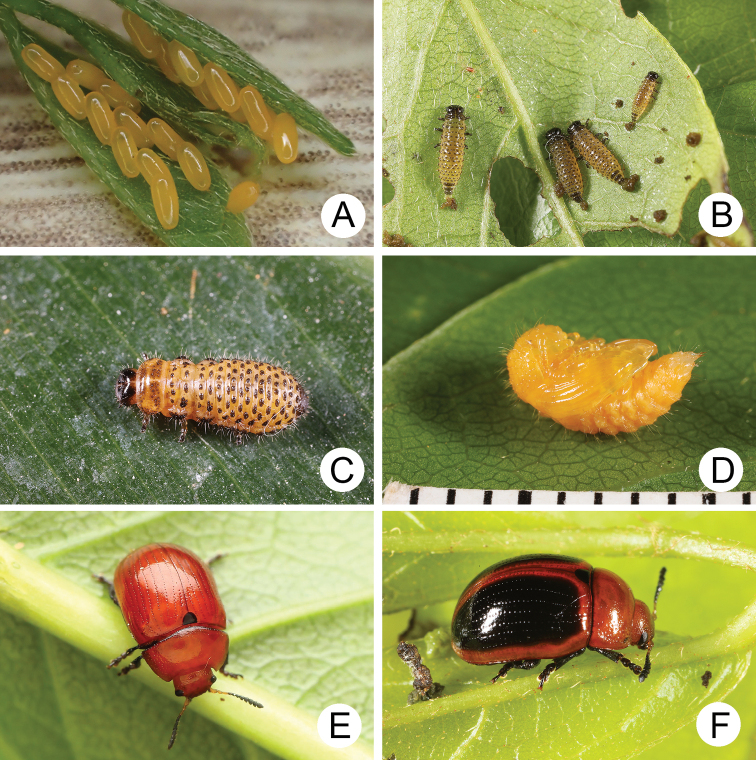
Natural history of Gonioctena (Brachyphytodecta) scutellaris Baly on host plant, *Calleryareticulata* (Fabaceae) **A** eggs **B** first- and second-instar larvae **C** fourth-instar larva **D** pupa **E** adult, typical form, from Daken (大坑), Taichung **F** adult, color variation, from Lienhuachih (蓮華池), Nantou.

Figs 8A–C , 9A–C , 10 , 11

Gonioctena scutellaris Baly, 1862: 27 (North China); Gressitt and Kimoto 1963: 364 (as synonym of G.fulva; misidentification).
Phytodecta (s. str.) scutellaris : Weise 1916: 181 (catalogue).
Phytodecta scutellaris : Chen 1934: 73.
Asiphytodecta scutellaris : Chen and Young 1941: 206 (key)
Gonioctena thoracica Baly, 1862: 27 (North China); Gressitt and Kimoto 1963: 364 (as synonym of G.fulva; misidentification). Syn. nov.
Phytodecta (s. str.) thoracicus : Weise 1916: 181 (catalogue).
Phytodecta thoracicus : Chen 1934: 73.
Asiphytodecta thoracicus : Chen and Young 1941: 206 (key)
Gonioctena dichroa Fairmaire, 1888: 153 (China: Jiangxi); synonymized with P.thoracicus by Chen (1934). Synonym confirmed
Phytodecta (s. str.) dichrous : Weise 1916: 181 (catalogue).
Phytodecta (Asiphytodecta) issikii Chûjô, 1958: 69 (Taiwan). Syn. nov.
Gonioctena (Asiphytodecta) issikii : Kimoto 1969: 22 (additional records in Taiwan)
Gonioctena (Brchyphytodecta) issikii : Gruev 1989: 54; Kimoto and Chu 1996: 53 (catalogue); Kimoto and Takizawa 1997: 369 (catalogue).
Gonioctena issikii : Takizawa et al. 1995: 7 (additional records in Taiwan)
Gonioctena (Brchyphytodecta) foochowensis Gruev, 1989: 53 (China: Fujian); Ge 2010: 66 (as synonym of G.fulva; misidentification). Syn. nov.

##### Types.

*Gonioctenascutellaris*. ***Lectotype*** ♂ (NMHUK, here designated to clarify its identity among other synonyms): “Type [h, w] // Type / H. T. [p, w, circle card with red border] // Baly Coll. [p, w] // Gonioctena / scutellaris / Baly / N: China [h, b]”.

*Gonioctenathoracica*. ***Lectotype*** ♂ (NMHUK, here designated for clarifying its identify with other synonyms): “(aedeagus preserved inside a small container) // Type [h, w] // Type / H. T. [p, w, circle card with red border] // Baly Coll. [p, w] // Gonioctena / thoracica / Baly / N: China [h, b] // Gonioctena / thoracica Baly [h] / det. H.W. Cho 2013 [p, w] // SYN- / TYPE [p, w, circle card with blue border]”. Paralectotype: 1♀ (NMHUK): “Type [h, w] // Baly Coll. [p, w] // SYN- / TYPE [p, w, circle card with blue border]”.

*Gonioctenadichroa*. ***Syntypes***: 1♂ (MNHN): “MUSEUM PARIS / KIANG-SI / A. DAVID 1875 [p, w] // 406 / 75 [h, w, circle label] // Gonioctena / dichroa / m. [h, w] // TYPE [p, w, red letters] // SYNTYPE [p, r] // Gonioctena / dichora Frm. / rev. M. Daccordi, 2005 [p, w] // Gonioctenadichora / Fiarmiare, 1888 / det. H.W. Cho 2014 [p, w] // MNHN, Pairs / EC14213 [p, w]”; 1♀ (MNHN): “MUSEUM PARIS / KIANG-SI / A. DAVID 1875 [p, w] // 406 / 75 [h, w, circle label] // 288 [h, green label] // SYNTYPE [p, r] // Gonioctenadichora / Fiarmiare, 1888 / det. H.W. Cho 2014 [p, w] // MNHN, Pairs / EC14214 [p, w]”. Fairmaire (1888) indicated that this species was described in Beijing (= Pekin) but both types actually were collected from Jiangxi (= Kiang-Si).

Phytodecta (Asiphytodecta) issikii. ***Holotype*** ♂ (TARI, original designation): “Baibara [h] (= Meiyuan, 眉原) / FORMOSA [p] / 24.III.1943 [h] / COL. [p] S. Issiki [h, w] // Phytodecta / issikii / / Chûjô [h] / DET. M. CHUJO [p, w] // Holo / Type [h, w; circle card with red letters and border but fade out] // 695 [p, w]”.

Gonioctena (Brchyphytodecta) foochowensis. ***Holotype*** ♀ (NMHUK, original designation): “CHINA / Foochow / C. R. Kelloqq [h, w] // Field No. [p] / 980 [h, w] // Phytodecta / sp [h] / Det. G. E. Bryant. [p] // Brit. Mus. / 198[p]1–315 [h, w] // Gonioctena / foochowensis / Gruev [p, w] // HOLOTYPE [p, r] // Gonioctenadichroa / Fairmaire, 1888 / det. H.W. Cho 2014 [p, w]”.

##### Other material (n = 150).

**China**. Fujian: 5♂, 8♀ (NMHUK), Foochow (福州), 1935–1938, leg. M. S. Yang; **Taiwan**. Nantou: 2♂, 1♀ (TARI), Lienhuachih (蓮華池), 28.IV.2016, leg. A. Li; Kinmen: Kinmen Island (金門): 14♂, 7♀ (TARI), Tsaitso trail (蔡厝古道), 20.IV.2021, leg. C.-F. Lee; 4♂, 1♀ (TARI), Taiwushan (太武山), 14.IV.2011, leg. Y.-J. Chang; 1♀ (TARI), same but with “6.V.2011”; 5♂, 2♀ (TARI), same but with “19.IV.2015”; 2♂, 4♀ (TARI), same but with “29–30.IV.2015”; 24♂, 23♀ (TARI), same locality, 8.IV.2021, leg. C.-F. Lee; 23♂, 27♀ (TARI), same but with “15.IV.2021”; Dadan Island (大膽島): 1♀ (TARI), 9.V.2016, leg. Y.-J. Chang; Matsu Islands: 1♂, 2♀ (TARI), Beigan Island (北竿), 1.V.2018, leg. H.-T. Fang; 1♂ (TARI), Nangan Island (南竿), 24.V.2009, leg. U. Ong.

##### Redescription.

Length 5.3–6.4 mm, width 3.5–4.0 mm. Body color (Fig. 8A, B) yellowish brown; antennomeres V–XI, legs, and scutellum black. Antennae (Fig. 10A) with antennomere III slender, IV–VI slightly swollen, VII moderately swollen, VIII-X strongly swollen, XI elongate oval, length ratios of antennomeres I–XI 1.0: 0.6: 0.6: 0.4: 0.4: 0.4: 0.5: 0.5: 0.5: 0.5: 0.9, length to width ratios of antennomeres I–XI 2.3: 1.9: 2.4: 1.7: 1.6: 1.0: 1.1: 1.1: 0.9: 0.9: 1.7. Pronotum 2.2–2.3× wider than long, lateral margins widest at base, convergent anteriorly, anterior angles strongly produced; anterior and lateral margins bordered, lateral margins barely visible in dorsal view; trichobothria absent on both anterior and posterior angles; disc covered with sparse fine punctures and mixed with finer punctures; both sides covered with much larger, denser punctures. Scutellum distinctly wider than long, narrowed posteriorly. Elytra 1.1–1.2× longer than wide; lateral margins slightly wider posteriorly, widest near middle; humeral calli well developed; disc covered with rather regular coarse punctures arranged into single stria; interspaces covered with fine, sparse punctures. Hind wing well developed. Aedeagus (Fig. 10B, C) with apical process extremely slender and elongate in dorsal view, 0.5× as long as aedeagus; lateral margins slightly narrowed in basal 1/3; strongly curved at basal 1/3 in lateral view; endophallic sclerite extremely elongate. Gonocoxae (Fig. 10D) wide, but apical margin irregular, with several long setae along apical margins. Ventrite VIII (Fig. 10E) transverse, with several long setae along outer margin. Receptacle of spermatheca (Fig. 10F) slightly swollen, not separated from pump; pump short and curved; sclerotized proximal spermathecal duct moderately short.

##### Variations.

Many adults possess black elytra with wide yellowish brown borders (Fig. 9A–C) that were described as *Gonioctenafoochowensis* and *G.issikii*. Some with the elytra entirely black were described as *G.dichroa* (Fig. 8C) and *G.thoracica*.

##### Diagnosis.

**Figure 12.**
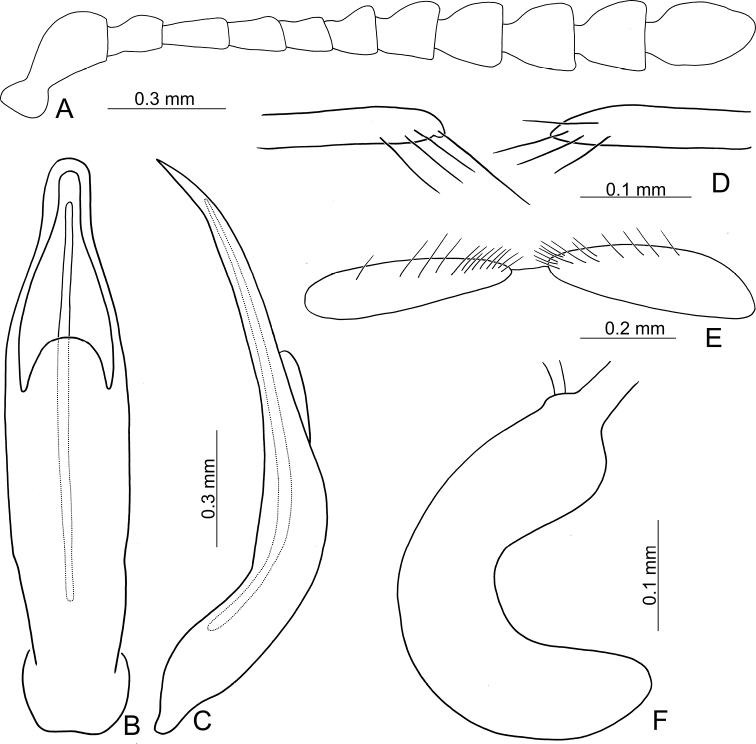
Diagnostic characters of Gonioctena (Brachyphytodecta) liui sp. nov. **A** antenna **B** aedeagus, dorsal view **C** aedeagus, lateral view **D** gonocoxae **E** abdominal ventrite VIII **F** spermatheca.

Gonioctena (Brachyphytodecta) scutellaris can be distinguished from the other consubgeneric species, G. (B.) liui sp. nov. by the following combination of the characters: yellowish brown head and pronotum (Figs 8A, C, 9A), thoracic and abdominal ventrites (Figs 8B, 9B) (black head and most parts of pronotum (Fig. 9D), thoracic and abdominal ventrites (Fig. 9E) in G. (B.) liui sp. nov.), extremely elongate apical process of aedeagus (Fig. 10B, C) (short, wide apical process of aedeagus in G. (B.) liui sp. nov. (Fig. 12B, C)), and wide gonocoxae covered with more setae (Fig. 10D) (narrow gonoxae covered with fewer setae in G. (B.) liui sp. nov. (Fig. 12D)).

##### Host plants.

Fabaceae: *Calleryareticulata* (Benth.) Schot (new record, present study)

##### Biology.

Gonioctena (Brachyphytodecta) scutellaris are presumed to be multivoltine during spring and females are oviparous. In Taiwan, this species seems rare due to unpredictable sprouting times of host plants. Mr. Alex Li (李志穎) collected some adults (Fig. 11F) and mature larvae on April 28, 2016, at Lienhuachih (蓮華池). However, we could not find this species around the same season in the following years because host plants had not sprouted. For example, we could not find sprouts of the host plants in late May 2021 at that location. The populations in small islands are more stable, where host plants began to sprout, and overwintered female laid eggs during middle of March 2021 in Kinmen Islands. They laid 12 eggs into two rows on very young leaves (Fig. 11A). Larvae hatched in three-four days. Early instar larvae (Fig. 11B) fed gregariously on young leaves but became solitary as they matured. The larval duration was nine days. Mature larvae (Fig. 11C) burrowed into soil and built underground chambers for pupation. Duration of the pupal stage (Fig. 11D) was nine days. The newly emerged adults (Fig. 11F) appeared during early April and females started to lay eggs after a week. In Jiangxi, China, females began to lay eggs first and then laid larvae in early April (Zhang and Shen 1984).

##### Remarks.

Chen (1934) regarded *G.dichroa* as synonym of *G.thoracica*. Gressitt and Kimoto (1963) synonymized *G.thoracica* and *G.scutellaris* with *G.fulva*. Ge (2010) treated *G.foochowensis* as a synonym of *G.fulva*. After examining aedeagi of these species, we concluded that *G.scutellaris* is a distinct species, which is removed from synonymy with *G.fulva*. Moreover, *G.dichora*, *G.thoracica*, and *G.foochowensis* are conspecific with *G.scutellaris*. We found that *G.issikii* is also a junior synonym of *G.scutellaris*. The typical color form (entirely yellowish brown elytra) is extremely rare in Kinmen and Matsu islands, and Taiwan. Only three specimens of such form were collected from Kinmen Island (金門島) and Dadan Island (大膽島). Many larvae were brought from Kinmen Island into the laboratory for rearing during March 2021. Only one specimen with the typical color form was among more than 50 adults reared from larvae. In addition, although no specimens of this form were collected from Taiwan, photographs of this form were taken at Darken (大坑), Taichng in May 1, 2020 (Fig. 11E) by Hsien Chung Liu (劉獻宗).

##### Distribution.

China, Taiwan, including Kinmen Islands (Kinmen Island 金門島, Dadan Island 大膽島) and Matsu Islands (Kaoteng Island 高登島, Beigan Island 北竿島, and Nangan Island 南竿島, new records).

#### ﻿ Gonioctena (Brachyphytodecta) liui sp. nov.

Taxon classificationAnimaliaColeopteraChrysomelidae

432D91A4-23A6-5330-A51F-E3236898EF42

https://zoobank.org/EECC59E7-265B-479E-9A7B-7C2EDCDBA4AE

Figs 9D–F , 12 , 13

Gonioctena issikii : Ge et al. 2007: 582 (aedeagus); Cho 2016: 95 (redescription). Misidentification

##### Types (n = 95).

***Holotype*** ♂ (TARI): TAIWAN. Nantou: Peitungyanshan (北東眼山), 3.VII.2014, leg. F.-S. Huang, 變葉新木薑子 (Neolitseaaciculatavar.variabillima) 噴霧 (fogging). ***Paratypes***. Nantou: 3♂, 4♀ (TARI), Lienhuachih (蓮華池), 7.V.2016, leg. C.-J. Liu; 5♂ (TARI), same but with “21.V.2016”, reared from larvae; 5♂, 5♀ (TARI), same locality, 10.V.2016, leg. H. Lee; 9♂, 9♀ (TARI), same but with “20.V.2017”; 5♂, 1♀ (TARI), same locality, 12.V.2016, leg. P.-H. Li; 2♀ (TARI), same locality, 22.III.2018, leg. J.-C. Chen; 3♂, 1♀ (TARI), same locality, 25.V.2019, leg. B.-H. Ho; 9♂, 15♀ (TARI), same locality, 20.IV.2020, leg. C.-F. Lee; 12♂, 7♀ (TARI), same but with “3.VI.2020”; 1♂, 3♀ (TARI), same locality, 16.IV.2021, leg. W.-C. Liao.

##### Description.

Length 5.3–6.1 mm, width 3.2–3.9 mm. Body color (Fig. 9D–F) blackish brown; antennomeres I–V, sides of pronotum and hypomeron yellowish brown; elytra with wide yellowish brown outer margins and suture. Antennae (Fig. 12A) with antennomere III slender, IV and V slightly swollen, VI and VII moderately swollen, VIII–X strongly swollen, XI elongate oval, length ratios of antennomeres I–XI 1.0: 0.5: 0.6: 0.5: 0.4: 0.4: 0.5: 0.6: 0.6: 0.6: 1.0, length to width ratios of antennomeres I–XI 2.1: 1.5: 2.2: 1.6: 1.5: 1.0: 1.0: 1.0: 1.1: 1.0: 1.8. Pronotum 2.3× wider than long, lateral margins widest at base, convergent anteriorly, anterior angles strongly produced; anterior and lateral margins bordered, lateral margins barely visible in dorsal view; trichobothria absent on both anterior and posterior angles; disc covered with sparse fine punctures and mixed with finer punctures; both sides covered with much larger, denser punctures. Scutellum distinctly wider than long, narrowed posteriorly. Elytra 1.3× longer than wide; lateral margins slightly wider posteriorly, widest near middle; humeral calli well developed; disc covered with rather regular coarse punctures arranged into single stria; interspaces covered with fine, sparse punctures. Hind wing well developed. Aedeagus (Fig. 12B. C) with apical process wide and short in dorsal view, 0.1× as long as aedeagus; lateral margins slightly narrowed in basal 1/3; strongly curved in lateral view; endophallic sclerite extremely elongate. Gonocoxae (Fig. 12D) narrow, but apically narrowed, with few long setae along apical margins. Ventrite VIII (Fig. 12E) transverse, with several long setae along outer margin. Receptacle of spermatheca (Fig. 12F) slightly swollen, not separated from pump; pump short and curved; sclerotized proximal spermathecal duct moderately short.

##### Diagnosis.

Gonioctena (Brachyphytodecta) liui sp. nov. can be distinguished from the other consubgeneric species, G. (B.) scutellaris, by the following combination of the characters: black head, most of pronotum (Fig. 9D), and thoracic and abdominal ventrites (Fig. 9E) (yellowish brown head and pronotum (Figs 8A, 9A), thoracic and abdominal ventrites (Figs 8B, 9B) in G. (B.) scutellaris), short, wide apical process of aedeagus (Fig. 12B, C) (extremely elongate apical process of aedeagus in G. (B.) scutellaris (Fig. 10B, C), and narrow gonocoxae covered with fewer setae (wide gonocoxae covered with more setae in G. (B.) scutellaris).

Although Ge et al. (2007) drew only the aedeagi in dorsal and lateral views when *G.issikii* was mentioned, it is easily identified as this new species. Cho (2016) redescibed *G.issikii* in detail but it actually fits perfectly this new species. The type specimen was not studied by these authors mentioned above.

##### Host plant.

Fabaceae: *Ormosiaformosana* Kanehira (new record, present study).

##### Biology.

**Figure 13.**
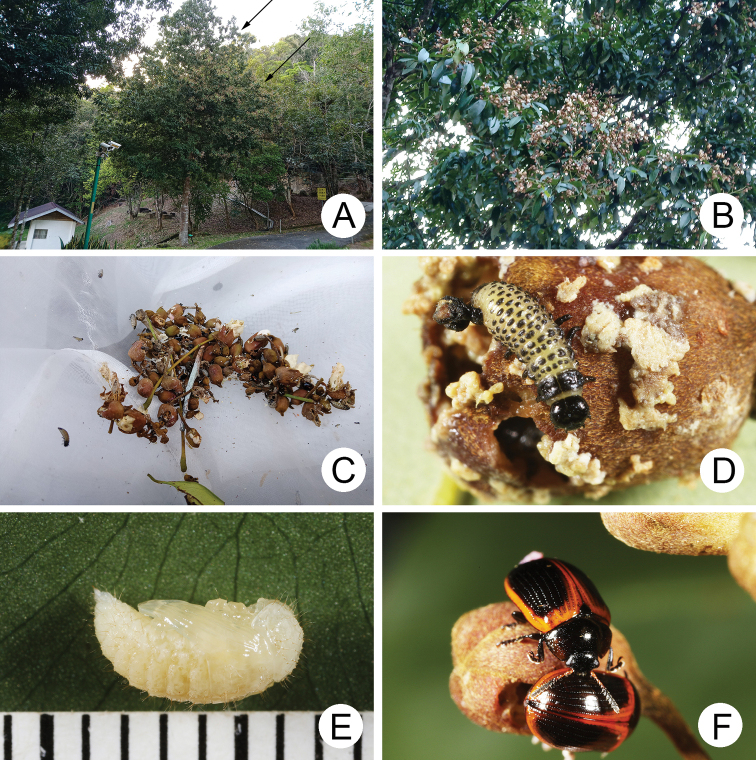
Natural history of Gonioctena (Brachyphytodecta) liui sp. nov. on host plant *Ormosiaformosana* (Fabaceae) **A** the whole tree, *Ormosiaformosana* (Fabaceae), the shape indicated by arrows **B** bloomed in April **C** flower buds and larvae collected with the sweeping net **D** fourth-instar larva burrowing into the soil and built underground chambers for pupation **E** pupa **F** adults.

Gonioctena (Brachyphytodecta) liui populations are presumed to be multivoltine during spring, and females are ovoviviparous. Host plants are one of only a few woody Fabaceae in Taiwan. They grow 7–9 m high (Fig. 13A). They started blooming during April (Fig. 13B). Most adults (Fig. 13F) and larvae were collected from flower buds (Fig. 13C). Females deposited larvae and the larvae (Fig. 13D) fed on internal tissues of the flower buds through holes chewed by adults. They preferred to feed on the flower buds rather than young sprouts. The larval duration was eight days. Mature larvae (Fig. 13D) burrowed into the soil and built underground chambers for pupation. The duration of the pupal stage (Fig. 13E) was nine days at room temperature. Flowering season of the host plant occurs from April to June (Huang and Ohashi 1993), but the blooming period for individual trees is less than a month.

##### Remarks.

The host plant is an endemic and rare species in Taiwan that is restricted to the central regions (Huang and Ohashi 1993). Few insects utilize it as a foodplant. The only species documented until now is a skipper, *Hasoraanurataiwana*Hsu et al. 2005 (Lepidoptera), which is monophagous on *Ormosiaformosana* Kanehira as a larval foodplant (Hsu et al. 2005).

##### Entomology.

The species name is dedicated to Mr. Cheng-Jr Liu (呂晟智) who collected types and discovered the host plant.

##### Distribution.

This new species is restricted to Central Taiwan.

#### ﻿ Gonioctena (s. str.) kamikawai

Taxon classificationAnimaliaColeopteraChrysomelidae

(Chûjô, 1958)

08A7B1F5-C917-56E2-B106-37414A88ED3E

**Figure 14.**
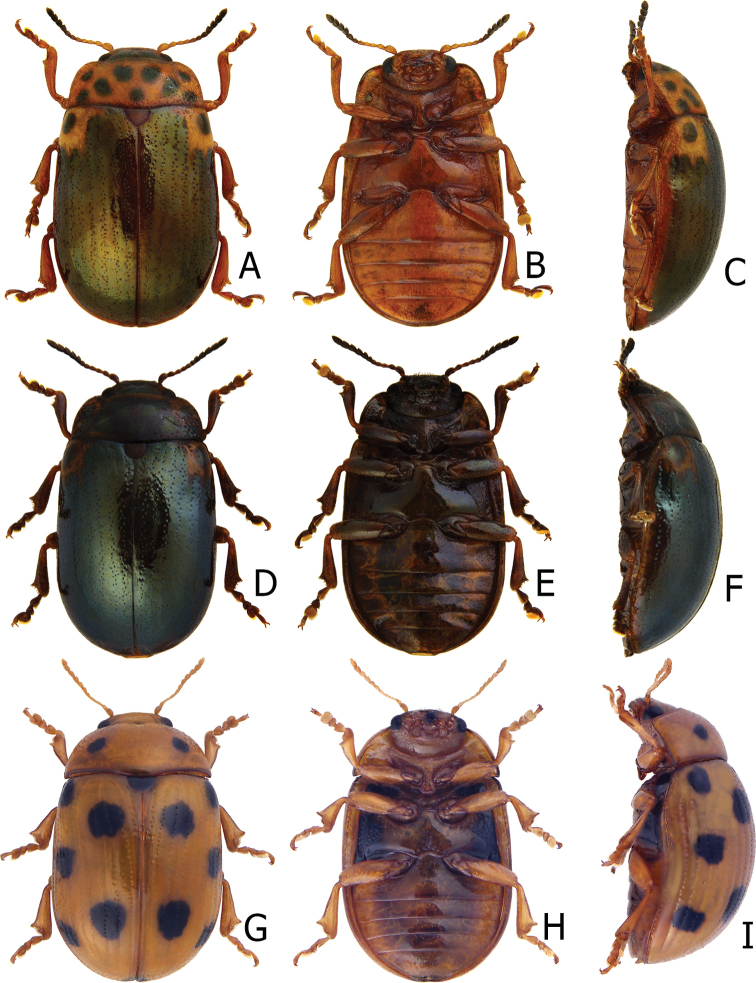
Habitus of Gonioctena(s. str.)kamikawai (Chûjô) and G. (Sinomela) osawai Kimoto **A**G.(s. str.)kamikawai, typical adult, dorsal view **B** ditto, ventral view **C** ditto, lateral view **D**G.(s. str.)kamikawai, darker adult, dorsal view **E** ditto, ventral view **F** ditto, lateral view **G**G. (Sinomela) osawai, dorsal view **H** ditto, ventral view **I** ditto, lateral view.

**Figure 15.**
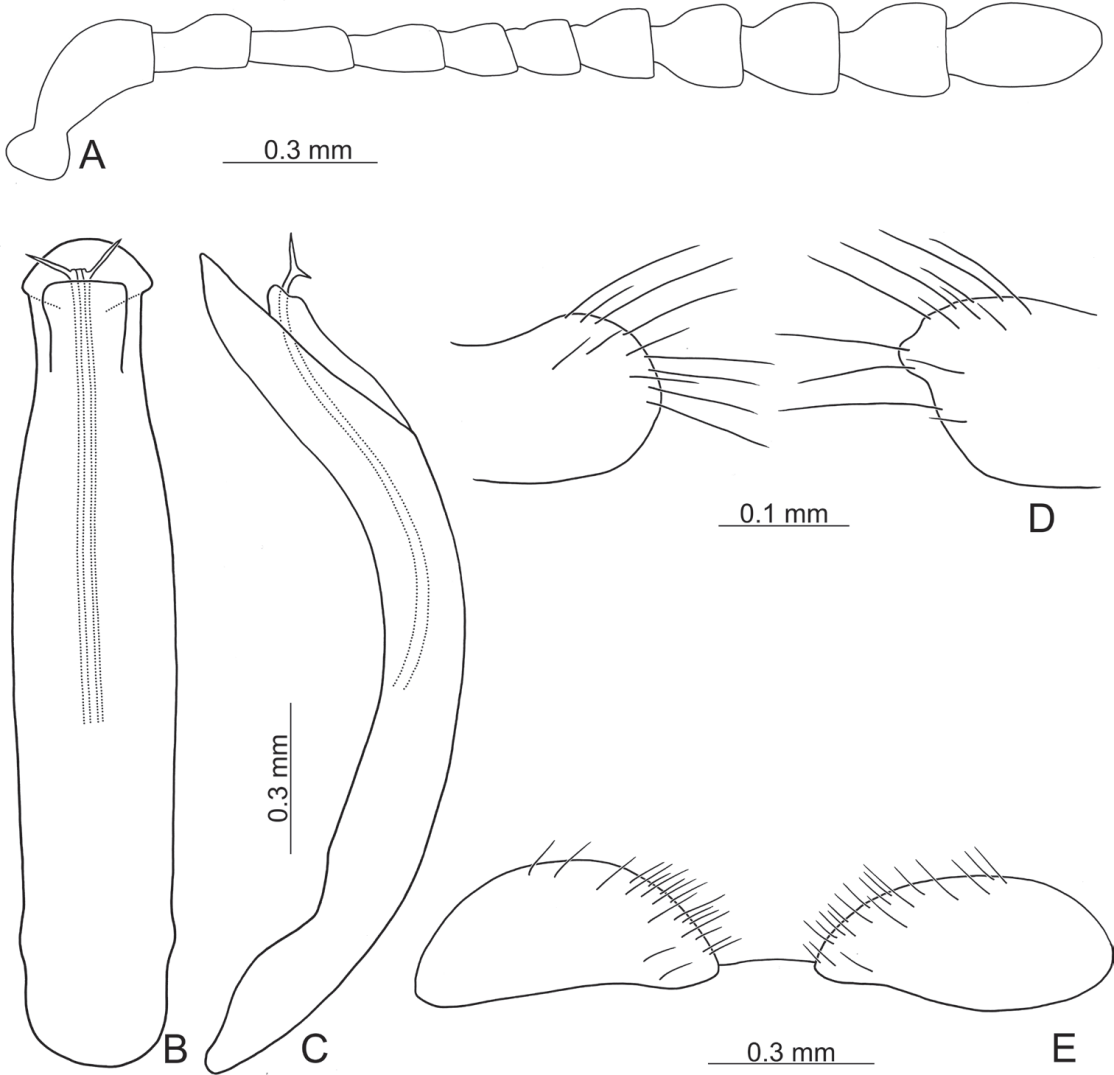
Diagnostic characters of Gonioctena(s. str.)kamikawai (Chûjô) **A** antenna **B** aedeagus, dorsal view **C** aedeagus, lateral view **D** gonocoxae **E** abdominal ventrite VIII.

**Figure 16.**
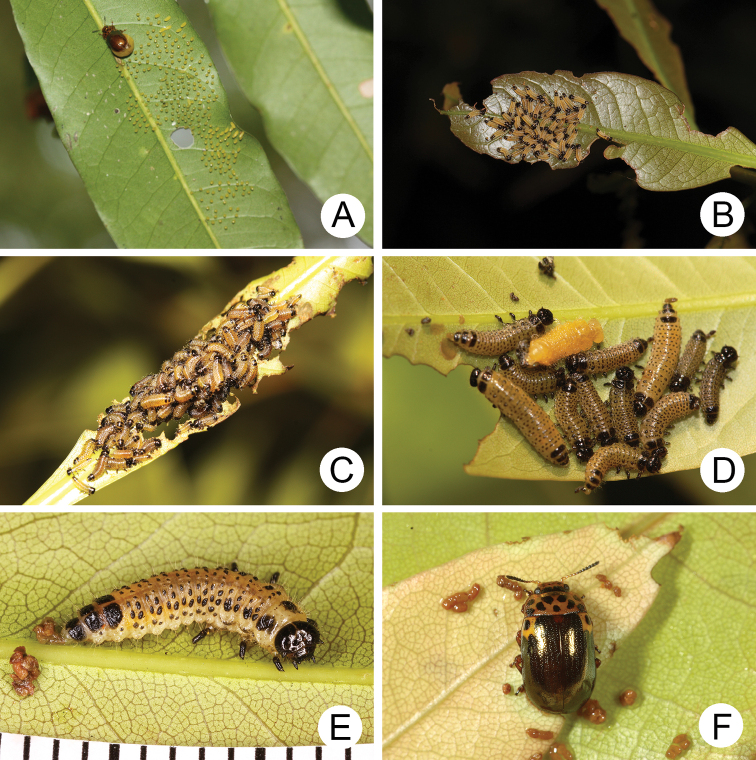
Natural history of Gonioctena(s. str.)kamikawai (Chûjô) on host plant, *Lithocarpusbrevicaudatus* (Fagaceae). **A** eggs **B** first-instar larvae **C** second-instar larvae **D** third-instar larva **E** fourth-instar larva **F** adult.

Figs 14A–F , 15 , 16

Phytodecta (s. str.) kamikawai Chûjô, 1958: 72 (Taiwan).
Gonioctena (s. str.) kamikawai : Kimoto 1969: 22 (additional records in Taiwan); Kimoto 1986: 56 (additional records in Taiwan); Kimoto and Chu 1996: 53 (catalogue); Kimoto and Takizawa 1997: 369 (catalogue); Wang et al. 1998: 49 (China: Fujian); Cho and Borowiec 2006: 179 (aedeagus); Kippenberg 2010: 433 (catalogue); Yang et al. 2015: 50 (catalogue); Cho 2016: 185 (redescription).
Gonioctena kamikawai : Kimoto 1987: 187 (additional records in Taiwan); Kimoto 1989: 247 (additional records in Taiwan); Takizawa et al. 1995: 7 (additional records in Taiwan).

##### Type.

***Holotype*** ♂ (TARI, original designation): “Hoorin [h] (= Fenglin, 鳳林) / FORMOSA [p] / 10.VIII.1934 [h] / COL. [p] M. Kamikawa [h, w] // Phytodecta / kamikawai / Chûjô [h] / DET. M. CHUJO [p, w] // Holo / Type [h, w; circle card with red letters and border but fade out] // 697 [p, w]”.

##### Other material (n = 77).

**Taiwan**. Hsinchu: 1♀ (TARI), Lupi (魯壁), 12.III.2009, leg. H. Lee; 1♂ (TARI), Mamei (馬美), 4.V.2008, leg. S.-F. Yu; 1♀ (TARI), Wufeng (五峰), 17.III.2009, leg. S.-F. Yu; Hualien: 1♂ (TARI), Jinma tunnel (金馬隧道), 5.V.2014, leg. J.-F. Tsai; 1♂, 1♂ (TARI), Pilu (碧綠), 17.V.2009, leg. M.-H. Tsou; 7♂, 2♀ (TARI), same locality, 1.VI.2009, leg. C.-F. Lee; 2♀ (TARI), same locality, 22.VI.2009, leg. U. Ong; Ilan: 1♀ (TARI), Fushan Botanical Park (福山植物園), 8.V.2008, leg. M.-H. Tsou; 1♂ (TARI), Suchi trail (四季林道), 19.V.2010, leg. H.-J. Chen; Kaohsiung: 2♂ (TARI), Chungchihkuan (中之關), 3.VII.2009, leg. S.-F. Yu; 1♂ (TARI), Erhchituan (二集團), 1.IV.2015, leg. B.-X. Guo; 2♂ (TARI), Tengchih (藤枝), 2–5.VI.2008, leg. C.-F. Lee; 1♂ (TARI), same locality, 2.X.2008, leg. M.-H. Tsou; 1♂ (TARI), same but with “26.V.2009”; 1♂ (TARI), same locality, 19.III.2013, leg. Y.-T. Chung; 1♂ (TARI), same locality, 8.II.2014, leg. W.-C. Liao; 2♂ (TARI), same but with “28.III.2015”; 1♂ (TARI), Tona trail (多納林道), 13.II.2013, leg. B.-X. Guo; 3♂ (TARI), same locality, 3.II.2013, leg. W.-C. Liao; Nantou: 1♂ (TARI), Chingching (清境), 6.IV.2010, leg. Y.-T. Wang; 1♂ (TARI), Kuantaoshan (關刀山), 19.IV.2014, leg. Y.-L. Lin; 1♂ (TARI), Tatachia (塔塔加), 9.VI.2009, leg. C.-F. Lee; 2♂ (TARI), same but with “27.IV.2010”; Pingtung: 2♀ (TARI), Tahanshan (大漢山), 8.V.2009, leg. U. Ong; 1♂ (TARI), same but with “1.VIII.2009”; 1♂, 1♀ (TARI), same locality, 24.IV.2013, leg. Y.-T. Chung; 1♂ (TARI), same but with “17.III.2014”; 1♂ (TARI), same but with “10.VI.2014”; 1♂ (TARI), same but with “24.II.2015”; 1♂ (TARI), same but with “14.II.2016”; 1♀ (TARI), same but with “9.IV.2018”; 2♂, 1♀ (TARI), same but with “29.VI.2018”; Taichung: 1♂ (TARI), Piluhsi (畢祿溪), 1.VII.2008, leg. M.-H. Tsou; 1♂ (TARI), Sungmao trail (松茂林道), 20.V.2012, leg. T.-H. Lee; 1♂ (TARI), Wuling (武陵), 30.VI.2008, leg. S.-F. Yu; Taipei: 1♀ (TARI), Manyuehyuan (滿月圓), 7.VI.2010, leg. C.-L. Chiang; Taitung: 1♂ (TARI), Liyuan (栗園), 23.VI.2010, leg. M.-H. Tsou; 1♂ (TARI), Hsiangyang (向陽), 28.III.2014, leg. W.-C. Huang; Taoyuan: 2♂ (TARI), Hsuanyuan (萱源), 21.IV.2010, leg. S.-F. Yu; 4♂ (TARI), Hsuehwunao (雪霧鬧), 2.IV.2011, leg. M.-H. Tsou; 2♀ (TARI), Paling (巴陵), 29–30.IV.2009, leg. M.-H. Tsou; 2♂, 1♀ (TARI), same but with “23.V.2009”; 4♂ (TARI), same but with “21.III.2010”; 1♀ (TARI), same but with “28.III.2010”; 2♂ (TARI), same but with “20.III.2011”; 1♂ (TARI), Tungyanshan (東眼山), 12.IV.2007, leg. H. Lee; 1♀ (TARI), same but with “2.V.2009”.

##### Redescription.

Length 5.5–7.3 mm, width 3.1–4.2 mm. Body color (Fig. 14A–C) yellowish brown; antennomeres VI–XI black; vertex with two large black spots; pronotum with nine black spots, arranged as follows: two pairs of black spots near apical margin, one pair near middle, the other pair near lateral margins; three black spots near basal margin, one pair near lateral margins, the other at middle; one pair of large black spots between one pair near middle and two spots near lateral margins; elytra copper brown, one pair of large black spots at humeral calli surrounding by yellowish brown border. Antennae (Fig. 15A) with antennomere III slender, IV–VI slightly swollen, VII and VIII moderately swollen, IX and X strongly swollen, XI elongate oval, length ratios of antennomeres I–XI 1.0: 0.5: 0.5: 0.5: 0.4: 0.3: 0.4: 0.5: 0.5: 0.6: 0.8, length to width ratios of antennomeres I–XI 2.6: 1.8: 2.3: 1.9: 1.4: 1.1: 1.1: 1.1: 1.1: 1.1: 2.0. Pronotum 2.3× wider than long, lateral margins widest at base, convergent anteriorly, anterior angles strongly produced; anterior and lateral margins bordered, lateral margins visible in dorsal view; trichobothria absent on anterior angles; disc covered with sparse fine punctures and mixed with finer punctures; both sides covered with much larger, denser punctures. Scutellum distinctly wider than long, narrowed posteriorly. Elytra 1.3–1.4× longer than wide; lateral margins slightly wider posteriorly, widest near middle, convergent posteriorly; humeral calli well developed; disc covered with irregular coarse punctures arranged into double striae; interspaces covered with fine, sparse punctures. Hind wing well developed. Aedeagus (Fig. 15B, C) with apical margin widely rounded; lateral margins slightly narrowed subapically; strongly curved in lateral view; endophallic sclerite extremely elongate, apex with one pair of pointed processes, basally membranous. Gonocoxae (Fig. 15D) wide, apical margins irregular, with sparse long setae along apical margins. Ventrite VIII (Fig. 15E) transverse and wide, with dense short setae along outer margin. Spermatheca reduced.

##### Variations.

In some adults the black spots on the vertex are combined. Some have enlarged black spots on the pronotum, sometimes connected, and darker antennomeres I–V, legs, and thoracic ventrites (Fig. 14D–F).

##### Diagnosis.

Gonioctena(s. str.)kamikawai is the only member of the subgenus Gonioctena in the Taiwan fauna. Thus it is easily recognized by its subgeneric character -- trichobothria present on posterior angles of pronotum (trichobothria absent on anterior and posterior angles of pronotum in the subgenera *Asiphytodecta* and *Brachyphytodecta*; trichobothira present on anterior and posterior angles of the pronotum in the subgenus Sinomela). In addition, this species is characterized by its color patterns, shape of aedeagus, and apical processes of endophallic sclerites.

##### Host plants.

Fagaceae: *Lithocarpushancei* (Benth.) Rehder (Lee and Cheng 2010), *L.brevicaudatus* (Skan) Hayata (present study); Betulaceae: *Alnusformosana* (Burkill) Makino (Lee and Cheng 2010). Adults fed on leaves of *Alnusformosana* in the laboratory but this was not observed in the field.

##### Biology.

Females laid more than 280 eggs at a time (Fig. 16A). Larvae hatched in 5–7 days. The early instar larvae (Fig. 16B–D) gregariously fed on young leaves but became solitary as mature larvae. Mature larvae (Fig. 16E) burrowed into the soil and built underground chambers for pupation. The duration of the pupal stage was ten days. Newly emerged adults (Fig. 16F) appeared after May and went into a resting stage after feeding. Three hibernating adults were found during May 2011 at Wulai (烏來), North Taiwan. They became active and fed on leaves on 15 January 2012. The following observations suggest that populations of Gonioctena(s. str.)kamikawai are multivoltine. Many larvae were found on 21 March 2010 at Paling (巴陵), North Taiwan; females were observed laying eggs on 10 May 2010, at Peitawushan (北大武山), South Taiwan. These adults were likely the first full generation of the season.

##### Distribution.

Endemic to Taiwan. This species is widespread in lowlands and mid-elevations.

#### ﻿ Gonioctena (Sinomela) nigroplagiata

Taxon classificationAnimaliaColeopteraChrysomelidae

Baly, 1862

D314E487-A901-50F6-8730-1A39E19BFD37

Figs 17 , 18 , 19

Gonioctena nigroplagata [sic!] Baly, 1862: 28 (Japan).
Phytodecta nigroplagiata : Jacoby 1885: 210 (catalogue).
Phytodecta (s. str.) nigroplagiatus : Weise 1916: 178 (catalogue).
Phytodecta nigroplagiatus : Winkler 1930: 1296 (catalogue); Chen 1934: 74 (China).
Phytodecta (Sinomela) nigroplagiatus : Chen 1935: 129 (catalogue); Chen 1936: 87 (catalogue).
Gonioctena (Sinomela) nigroplagiatus : Chûjô and Kimoto 1961 (catalogue).
Gonioctena (Sinomela) nigroplagiata : Gressitt and Kimoto 1963: 366 (catalogue); Kimoto 1964: 280 (catalogue); Takizawa 1976: 464 (larva); Takizawa 2007: 45 (aedeagus and color polymorphism); Kippenberg 2010: 436 (catalogue); Yang et al. 2015: 55 (catalogue); Cho 2016: 294 (redescription).
Phytodecta robusta Jacoby, 1885: 209 (Japan); synonymized by Chûjô and Kimoto (1961). Synonym confirmed.
Phytodecta (s. str.) robustus : Weise 1916: 181 (catalogue).
Phytodecta robustus : Winkler 1930: 1296 (catalogue).
Phytodecta (Sinomela) nigroplagiatus var. robustus : Chen 1935: 129 (catalogue); Chen 1936: 87 (catalogue).

##### Types.

*Gonioctenanigroplagiata*. ***Lectotype*** ♀ (here designated to clarify identity relative to *G.robusta*, NMHUK): “Type [h, w] // Type / H. T. [p, w, circle card with red border] // Baly Coll. [p, w] // Gonioctena / nigroplagiata / Baly / Japan [p, b] // Gonioctena / (Sinomela) / nigroplagiata / (Baly) [h] / Det. S. GE 2004 [p, w]”. Paralectotype: 1♀ (NMHUK): “Type [h, w] // Baly Coll. [p, w] // SYN- / TYPE [p, w, circle card with blue border]”.

*Phytodectarobusta*. ***Lectotype*** ♂ (Here designated for to clarify identity relative to *G.nigroplagiata*, NMHUK): “Japan / Lewis [h, w] // SYN- / TYPE [p, w, circle card with blue border] // Jacoby Coll. / 1909–28a. [p, w] // robusta Jac [h, b]”. Paralectotype: 1♀ (TARI): “Hiogo / JAPAN / 8.VI.1881 / Col. G. Lewis [h, w] // CO / Type [p, w, circle card with yellow letters and border] // Phytodecta / robustus / Jacoby [h] / DET. M. CHUJO [p, w] // Phytodecta / robusta Jac [h] / Det. T. Shiraki [p, w] // 1636 [p, w]”.

##### Other material (n = 95).

**Taiwan**. Matsu islands: 17♂, 17♀ (TARI), Nangan (南竿), 7.VI.2017, leg. T.-C. Chen; 23♂, 35♀ (TARI), same locality, 8.VI.2017, leg. Y.-L. Liu; 1♀ (TARI), same locality, 28.VI.2017, leg. H.-T. Fang; 1♂, 1♀ (TARI), Beigan (北竿), 18.IV.2020, C.-H. Tsieh.

##### Redescription.

**Figure 17.**
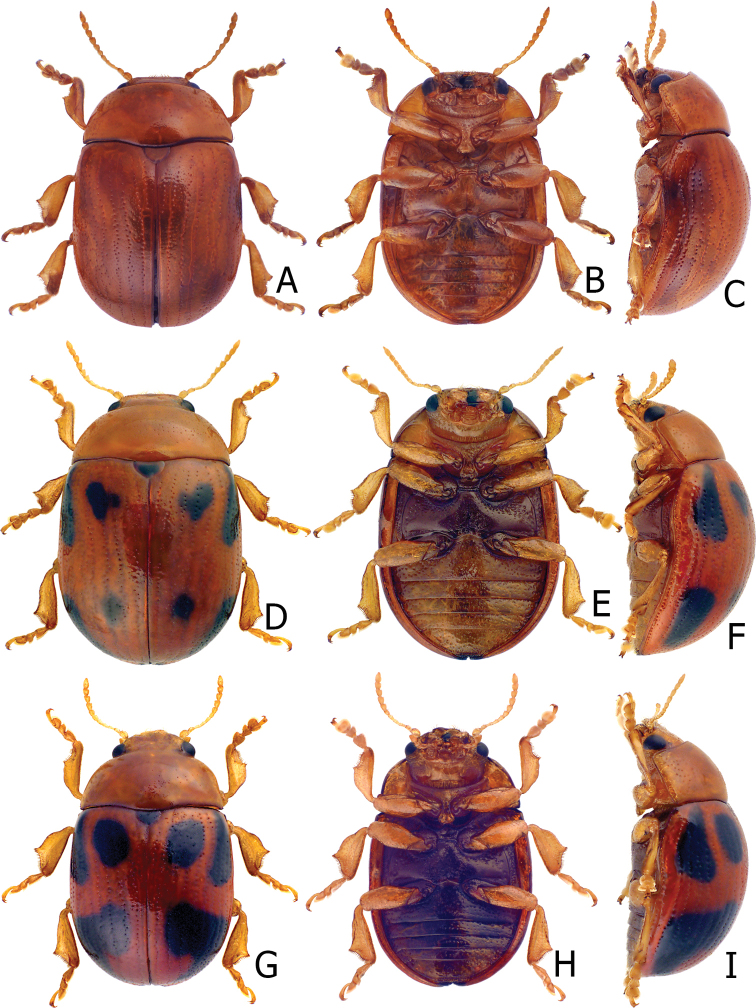
Habitus of Gonioctena (Sinomela) nigroplagiata Baly **A** pale individual, dorsal view **B** ditto, ventral view **C** ditto, lateral view **D** intermediate individual, dorsal view **E** ditto, ventral view **F** ditto, lateral view **G** individual with predominant black spots on the elytra, dorsal view **H** ditto, ventral view **I** ditto, lateral view.

**Figure 18.**
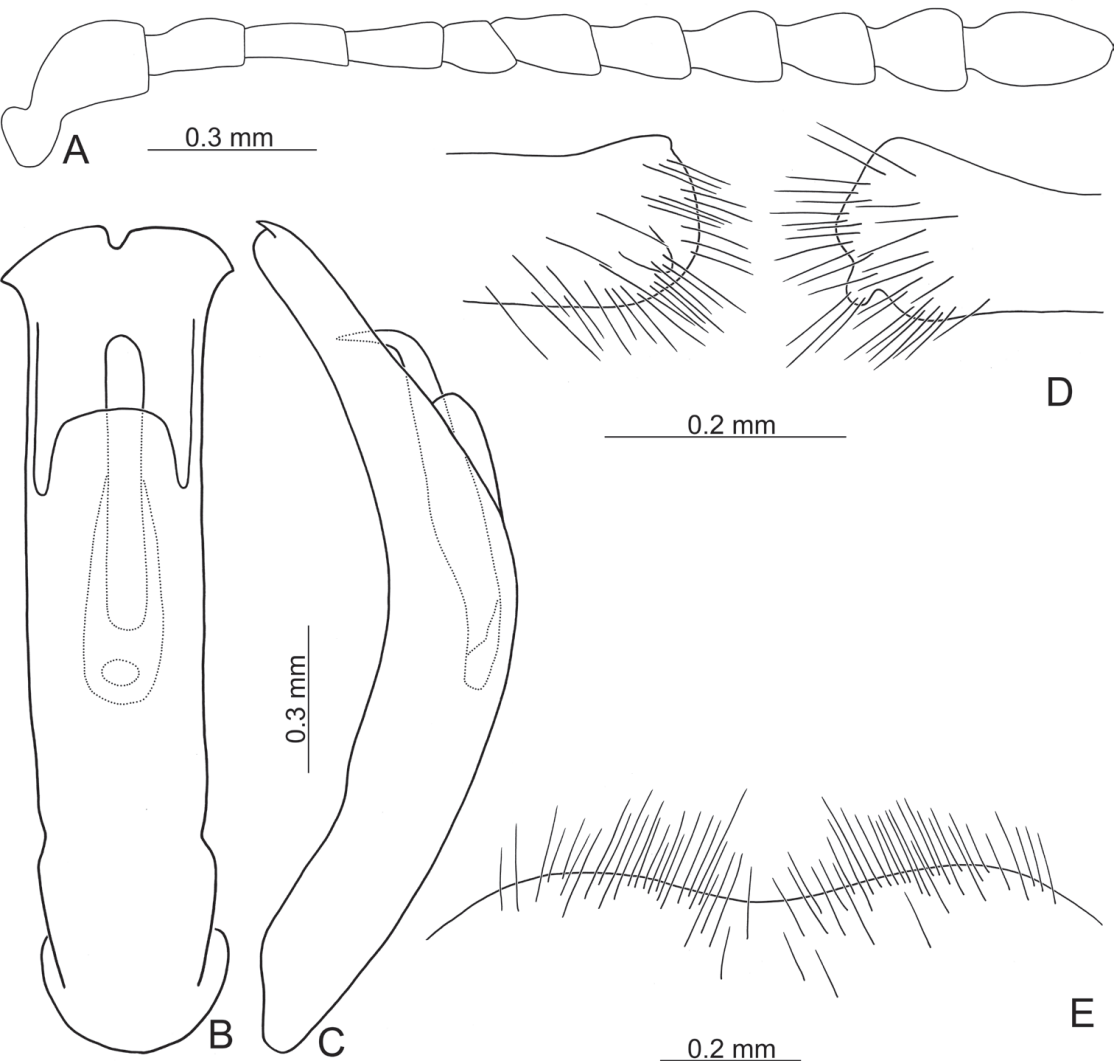
Diagnostic characters of Gonioctena (Sinomela) nigroplagiata Baly **A** antenna **B** aedeagus, dorsal view **C** aedeagus, lateral view **D** gonocoxae **E** abdominal ventrite VIII.

Length 6.1–7.3 mm, width 3.8–4.7 mm. Body color (Fig. 17G–I) yellowish brown; scutellum black; elytra with three pairs of black spots arranged as follows: posterior pair largest at apical 1/3, transverse, from near suture to near lateral margins, extending posteriorly near lateral margins; two pairs of black spots near basal margin, one oval to oblong pair near suture, the other on humeral calli, triangular, anteriorly narrowed. Meso- and metathorcic and abdominal ventrites darker. Antennae (Fig. 18A) with antennomere III–IV slender, V–VI slightly swollen, VII–X moderately swollen, XI elongate oval, length ratios of antennomeres I–XI 1.0: 0.6: 0.6: 0.6: 0.3: 0.5: 0.5: 0.5: 0.5: 0.5: 0.9, length to width ratios of antennomeres I–XI 2.2: 1.9: 2.7: 2.1: 1.0: 1.5: 1.5: 1.4: 1.3: 1.1: 2.0. Pronotum 2.3–2.4× wider than long, lateral margins widest at base, convergent anteriorly, anterior angles strongly produced; anterior and lateral margins bordered, lateral margins barely visible in dorsal view; trichobothria present on anterior and posterior angles; disc covered with sparse fine punctures and mixed with finer punctures; both sides covered with much larger, denser punctures. Scutellum distinctly wider than long, narrowed posteriorly. Elytra 1.1× longer than wide; lateral margins slightly wider posteriorly, widest near middle, convergent posteriorly; humeral calli well developed; disc covered with regular coarse punctures arranged into single stria; interspaces covered with fine, sparse punctures. Hind wing well developed. Aedeagus (Fig. 18B, C) with apical margin widely rounded and medially notched, anterior angles acutely hooked, lateral margins slightly narrowed at basal 1/3; moderately curved in lateral view; endophallic sclerite short, apex narrowly rounded in dorsal view, acute process in lateral view. Gonocoxae (Fig. 18D) wide, apical margins irregular, with dense long setae along apical margins. Ventrite VIII (Fig. 18E) transverse and membranous, with dense long setae along outer margin. Spermatheca reduced.

##### Variations.

Black areas on dorsum and venter are reduced to different degrees in various individuals. Some adults have smaller black spots arranged into four pairs on the elytra, with only the central part of the scutellum blackish brown, and only the meso- and metathoracic ventrites darker (Fig. 17D–F). Pale adults have entirely yellowish brown bodies (Fig. 17A–C).

##### Diagnosis.

Gonioctena (Sinomela) nigroplagiata is easily separated from the other consubgeneric species, G. (S.) osawai by the following combination of the characters: no black spots on the dorsum, none or three or four pairs of black spots on the elytra (Figs 17) (one pair of black spots on pronotum and five pairs of black spots on elytra in G. (S.) osawai (Fig. 14G); wide antennomere V, width subequal to length (Fig. 18A) (elongate antennomere V, more than 2.0× longer than wide in G. (S.) osawai (Fig. 20A)); narrowly rounded apex of endophallic sclerites (Fig. 18B) (bifurcate and asymmetrical apices of endophallic sclerites in G. (S.) osawai (Fig. 20B)); wide gonocoxae covered with more setae (Fig. 18D) (narrow gonocoxae covered with fewer setae in G. (S.) osawai (Fig. 20D)); membranous ventrite VII with dense setae along apical margin (Fig. 18E) (slightly sclerotized ventrites VIII with dense setae only on apices in G. (S.) osawai (Fig. 20E)).

##### Host plants.

**Figure 19.**
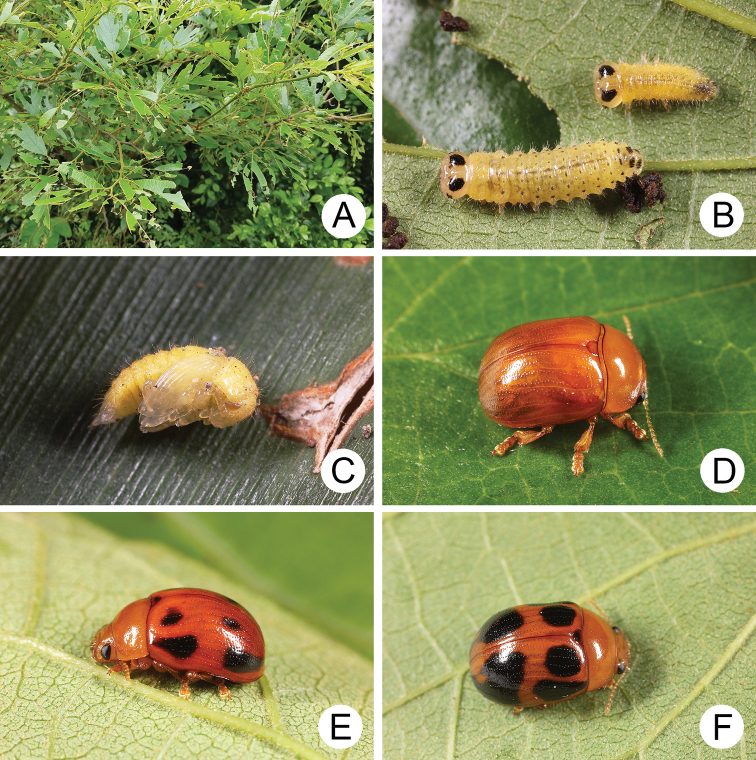
Natural history of Gonioctena (Sinomela) nigroplagiata Baly on host plant, *Celtissinensis* (Cannabaceae) **A** leaves of the host plant fed by a lot of larvae **B** fourth-instar larva **C** pupa **D** pale adult **E** intermediate adult **F** adult with predominant black spots on the elytra.

Cannabaceae: *Celtissinensis* Pers. (Chûjô and Kimoto 1961; present study) (Fig. 19A).

##### Biology.

A mass outbreak of adults of this species occurred on Matsu Islands during June 2017. The large numbers of beetles made local citizens nervous and made the news. A number of specimens were sent to the authors for identification. Mr. Hua-Te Fang (方華德) collected larvae (Fig. 19B) on 15 April 2021 at the locality. Mature larvae burrowed into soil and built underground chambers for pupation (Fig. 19C) on 22 April. The first adults emerged from soil on 2 May and were entirely yellowish brown (Fig. 19D). Black spots appeared after three days in some individuals and become stable within five days (Fig. 19E, F). No adults were observed in the field by Mr. Fang on 11 May, 29 September, and 21 October. This species is presumed to be univoltine, and larvae are active only during spring.

##### Remarks.

The original spelling in the original description (Baly 1862) is incorrectly printed as “*Gonioctenanigroplagata*”. It is obviously a typographical error and the correct spelling is “*Gonioctenanigroplagiata*”.

##### Distribution.

China, Japan, Taiwan (only on Matsu Islands, including Beigan Island北竿島and Nangan Island 南竿島).

#### ﻿ Gonioctena (Sinomela) osawai

Taxon classificationAnimaliaColeopteraChrysomelidae

Kimoto, 1996

48474EC3-47BA-5A27-A614-B4252EC7244B

**Figure 20.**
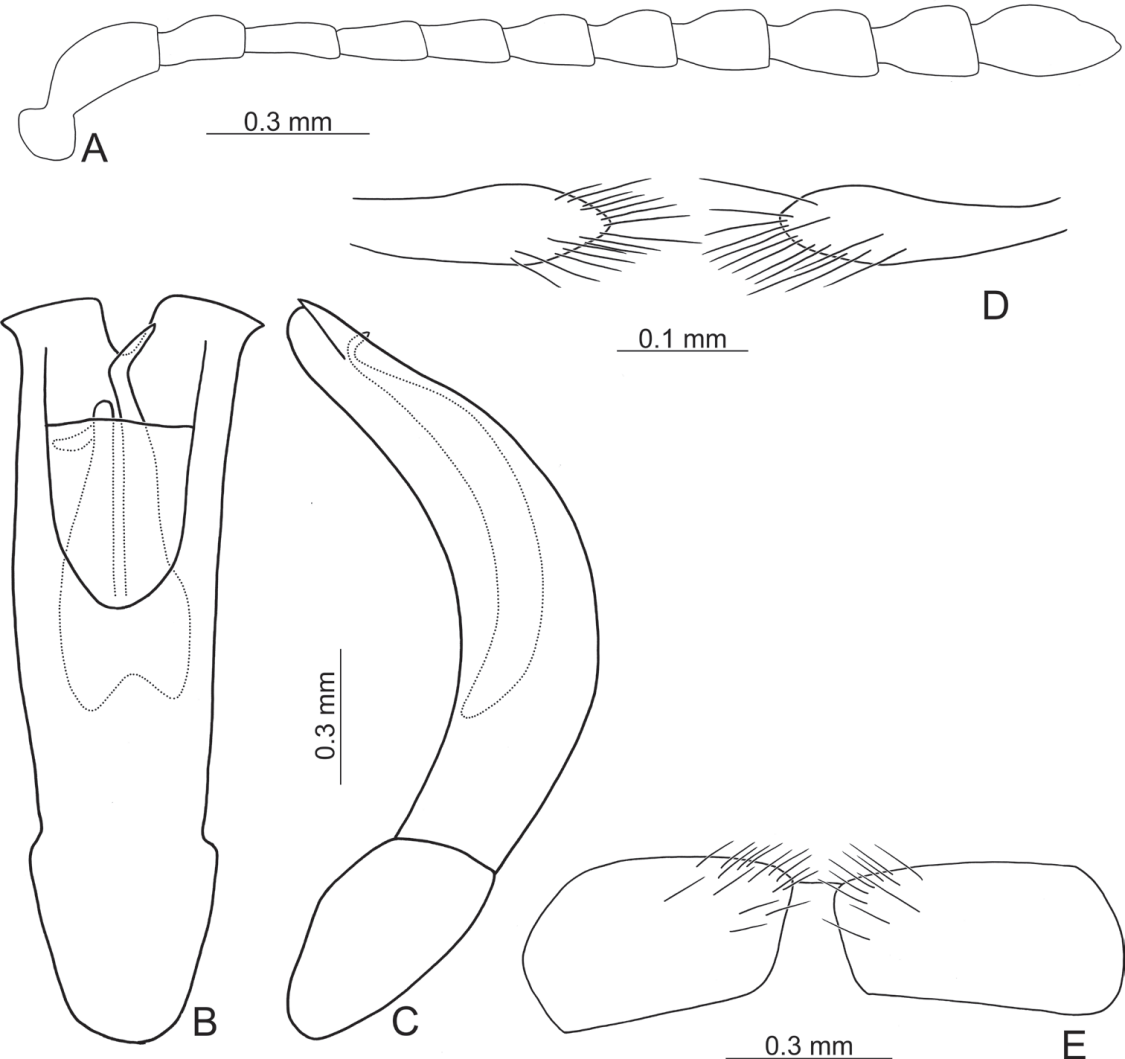
Diagnostic characters of Gonioctena (Sinomela) osawai Kimoto **A** antenna, **B** aedeagus, dorsal view **C** aedeagus, lateral view **D** gonocoxae **E** abdominal ventrite VIII.

**Figure 21.**
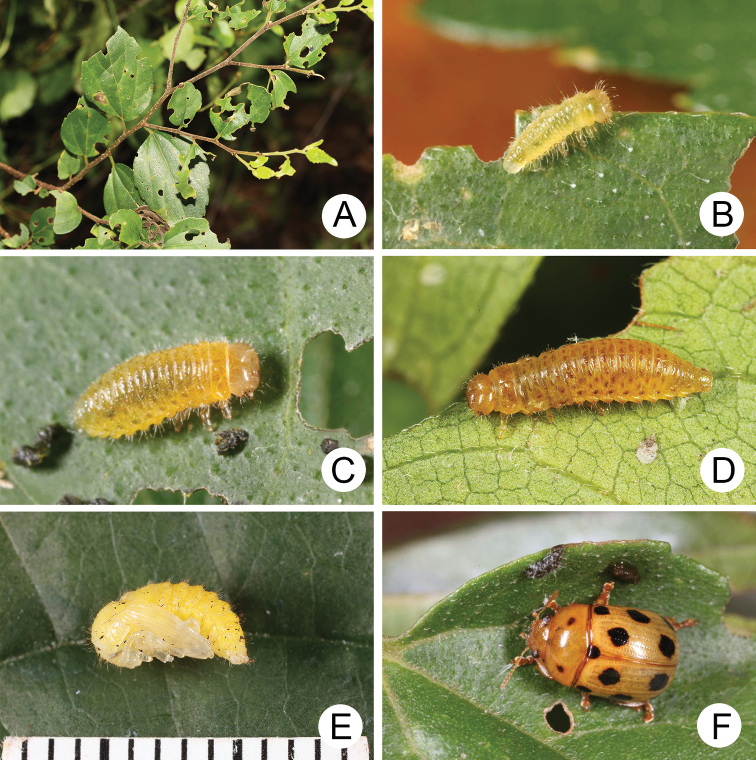
Natural history of Gonioctena (Sinomela) osawai Kimoto on host plant, *Celtisbiondii* (Cannabaceae) **A** host plant, *Celtisbiondii***B** first-instar larvae **C** second-instar larvae **D** third-instar larva **E** pupa **F** adult.

Figs 14G–I , 20 , 21

Gonioctena (Sinomela) osawai Kimoto, 1996: 29 (Taiwan); Kimoto and Takizawa 1997: 370 (catalogue); Kippenberg 2010: 436 (catalogue); Yang et al. 2015: 55 (catalogue).

##### Type.

***Holotype*** (sex undetermined, KMNH): “Nr Liukuei [p] (六龜) 溪南山 [h] (Chinanshan) / Kaohsiung Hs. Taiwan [p] / 20 I[h]V 19[p]91[h] / W. Chen leg (Osawa) [p, w] // PHOTO [p, r] // HOLOTYPE [p, r] // Gonioctena / osawai / Kimoto, n. sp [h] / Det. S. Kimoto, 19 [p, w] // 2001822IR02 [p, w] // KMNHIR200,093 [p, w]”. This holotype was deposited originally at the Biohistory Research Hall, Takatsuki, Osaka (Kimoto 1996). Now it is transferred to the KMNH.

##### Other material (n = 36).

**Taiwan**. Kaohsiung: 1♂ (TARI), Hsiaokuanshan (小關山), 15.V.2016, leg. B.-X. Guo; Pingtung: 2♀ (TARI), Chichia (七佳), 27.VII.2018, leg. Y.-T. Chung; 1♂, 1♀ (TARI), same but with “17.VII.2019”; 2♂, 2♀ (TARI), same locality, 18.VII.2019, leg. B.-X. Guo; 1♀ (TARI), Lilungshan (里龍山), 4.VIII.2009, leg. J.-C. Chen; 2♂ (TARI), same but with “15.VIII.2009”; 1♂ (TARI), same locality and date, le.g S.-F. Yu; 2♂ (TARI), same but with “leg. M.-H. Tsou; 6♂, 2♀ (TARI), Laochichia (老七佳), 15.VII.2021, leg. Y.-T. Chung; 5♂, 6♀ (TARI), same but with “30.VIII.2021”; 1♂ (TARI), Tahanshan (大漢山), 30.V.2012, leg. J.-C. Chen; 1♀ (TARI), same locality, 4.VI.2016, leg. Y.-F. Hsu; 1♂ (TARI), same locality, 14.IV.2020, leg. Y.-T. Chung;

##### Redescription.

Length 5.6–6.7 mm, width 3.7–4.7 mm. Body color (Fig. 14G–I) yellowish brown; vertex with one small black spot near center; scutellum black; pronotum with one pair of black spots at sides; elytra with five pairs of black spots, arranged as follows: three pairs near sides, one pair on humeral calli, one pair at middle, the other at apical 1/4; two pairs near suture, one pair at basal 1/3, the other at apical 1/3. Meso- and metathoracic ventrites black. Antennae (Fig. 20A) with antennomere III and IV slender, V and VI slightly swollen, VII–X moderately swollen, XI elongate oval, length ratios of antennomeres I–XI 1.0: 0.5: 0.5: 0.5: 0.5: 0.5: 0.5: 0.6: 0.6: 0.6: 0.9, length to width ratios of antennomeres I–XI 2.8: 1.9: 2.7: 2.2: 2.1: 1.8: 1.5: 1.6: 1.6: 1.4: 2.2. Pronotum 2.0–2.2× wider than long, lateral margins widest at base, convergent anteriorly, anterior angles strongly produced. Anterior and lateral margins bordered, lateral margins barely visible in dorsal view. Trichobothria present on anterior and posterior angles. Disc covered with sparse fine punctures mixed with finer punctures; both sides covered with much larger, denser punctures. Scutellum distinctly wider than long, narrowed posteriorly. Elytra 1.2× longer than wide; lateral margins slightly wider posteriorly, widest near middle, convergent posteriorly; humeral calli well developed; disc covered with regular coarse punctures arranged into single stria; interspaces covered with fine, sparse punctures. Hind wing well developed. Aedeagus (Fig. 20B, C) with apical margin widely rounded and medially notched, anterior angles acutely hooked, lateral margins slightly narrowed at basal 1/3; moderately curved in lateral view; endophallic sclerite short, apically bifurcate, right apical process twisted anteriorly and obliquely in dorsal view, recurved in lateral view; left apical process recurved and turned left. Gonocoxae (Fig. 20D) slender, apical margins apically narrowed, with dense long setae along outer and apical margins. Ventrite VIII (Fig. 20E) transverse, with dense long setae at apical areas. Spermatheca reduced.

##### Diagnosis.

Gonioctena (Sinomela) osawai is easily separated from the other consubgeneric species, G. (S.) nigroplagiata by the following combination of the characters: one pair of black spots on pronotum and five pairs of black spots on the elytra (Fig. 14G) (no black spots on the dorsum, or three or four pairs of black spots on the elytra in G. (S.) nigroplagiata (Fig. 17)); elongate antennomere V, more than 2.0× longer than wide (Fig. 20A) (wide antennomere V, width subequal to length in G. (S.) nigroplagiata (Fig. 18A)); bifurcate and asymmetric apices of endophallic sclerites (Fig. 20B) (narrowly rounded apex of endophallic sclerites in G. (S.) nigroplagiata (Fig. 18B)); narrow gonocoxae covered with fewer setae (Fig. 20D) (wide gonocoxae covered with more setae n in G. (S.) nigroplagiata (Fig. 18D)); slightly sclertozied ventrites VIII with dense setae only on apices (Fig. 20E) (membranous ventrite VII with dense setae along apical margin in G. (S.) nigroplagiata (Fig. 18E)).

##### Host plants.

Cannabaceae: *Celtisbiondii* Pamp. (Fig. 21A; present study).

##### Biology.

Gonioctena (Sinomela) osawai populations are presumed to be univoltine during summer and females are ovoviviparous. Sprouting season of the host plant is during summer. Adults (Fig. 21F) were found and brought into the laboratory on 15 July 2021. Females deposited larvae (Fig. 21B) the following day. The larval duration (Fig. 21B–D) was 12 days. Mature larvae (Fig. 21D) burrowed into the soil and built underground chambers for pupation. The duration of the pupal stage (Fig. 21E) was 13 days.

##### Distribution.

South Taiwan (Kaohsiung and Pingtung counties).

### ﻿Key to Taiwanese species of *Gonioctena*

**Table:** 

1	Trichobothria absent on anterior and posterior angles of pronotum	**2**
–	Trichobothria present on posterior angles, or on anterior and posterior of angles of pronotum	**5**
2	Punctures on elytra irregularly arranged or entirely confused	(subgenus Asiphytodecta)	**3**
–	Punctures on elytra regularly arranged into longitudinal rows	(subgenus Brachyphytodecta)	**4**
3	Punctures on elytra entirely confused; scutellum black; three black spots on pronotum; ten black spots on elytra, two black spots on suture, one at apical 1/3 and the other near apices (Fig. 2D)	**G. (A.) tredecimmaculata**
–	Punctures on elytra irregularly arranged into longitudinal rows; scutellum yellowish brown; no black spots on pronotum; eleven black spots on elytra, three black spots on suture, one at basal 1/3, median one at apical 1/3, the other at apices (Fig. 2A)	**G. (A.) subgeminata**
4	Head, pronotum, thoracic and abdominal ventrites yellowish brown (Figs 8A–C, 9A–C)	**G. (B.) scutellaris**
–	Head, most parts of pronotum, thoracic and abdominal ventrites black (Fig. 9D–F)	**G. (B.) liui sp. nov.**
5	Trichobothria present on posterior angles (subgenus Gonioctena); nine black spots on pronotum; elytra copper brown, one pair of large black spots at humeral calli surrounding by yellowish brown border (Fig. 14A–C)	**G. (G.) kamikawai**
–	Trichobothria present on anterior and posterior angles (subgenus Sinomela); no or one pair of black spots on pronotum; elytra yellowish brown, without black spots, or with three to five pairs of black spots	**6**
6	Antennomere V elongate, more than 2.0× longer than wide; one pairs of black spots on pronotum; five pairs of black spots on elytra (Fig. 14G–I)	**G. (S.) osawai**
–	Antennomere V wide, as long as wide; none black spots on pronotum; none or three to five pairs of black spots on elytra (Fig. 17)	**G. (S.) nigroplagiata**

#### Species excluded from the Taiwanese fauna

##### ﻿ Gonioctena (Brachyphytodecta) fulva

Taxon classificationAnimaliaColeopteraChrysomelidae

(Motschulsky, 1861)

67941529-ED42-5AFA-A5A4-E164365834BE

Figs 8D–F , 22

Spartophila fulva Motschulsky, 1861: 41 (China: Heilongjiang).
Phytodecta fulva : Kraatz 1879: 136 (catalogue of Heilongjiang); Heyden 1887: 262 (Korea); Weise 1893: 1127 (key); Chûjô 1941: 74 (Korea).
Phytodecta (s. str.) fulvus : Weise 1916: 177 (Catalogue); Chen 1935: 127 (catalogue)
Phytodecta fulvus : Winkler 1930: 1294 (catalogue).
Gonioctena (Brachyphytodecta) fulva : Gressitt & Kimoto, 1963: 364 (China: Jilin, Sichuan, Fujian, Zhejiang, Russia: Siberia); Takizawa 1980: 5 (Korea); Lee and An 2001: 101 (Korea); Cho and Lee 2008: 105 (Korea); Kippenberg 2010: 432 (China: Guandong, Hubei, Jiangxi; Russia: East Siberia, Far East); Yang et al. 2015: 52 (China: Hebei, Shanxi, Jiangsu, Hunan).
Gonioctena fulva : Takizawa 1985: 6 (Korea); Sergeev 2019: 14 (Russia: Popov Island).

###### Types.

***Lectotype*** ♀ (here designated, ZMUM): “Type [h, w] // Amur. [p, r] // Spartophila / fulva Motsch / Amur. mu. [h, w] // LECTOTYPUS / Spartophilafulva / Motschulsky, 1861 / des. H.W. Cho 2014 [p, r] // (Russian letters) // No ZMMU**Col 03055** / Zool. Mus. Mosq. Univ. / (Mosquae, ROSSIA) / ex coll. **V. I. Motschulsky** [p, pink label]”. The paralecrotype it is badly damaged, and its internal parts are destroyed by dermestids: “Spartophila / fulva Motsch. / fl. Amur. [h, w] // PARALECTOTYPUS / Spartophilafulva / Motschulsky, 1861 / des. H.W. Cho 2014 [p, r]. The lecotype and paralectotype were designated in Cho’s dissertation (2016), but not published.

###### Other material (n = 17).

**China**. Heilongjiang: 1♂ (NMHUK), Erlungshan, 29.V.1966, leg. P. M. Hammond; 1♀ (NMHUK), Habrin City (哈爾濱市), 10.VI.1950; 5♂, 1♀ (NMHUK), same but with “22.VI.1952”; 1♂, 1♀ (NMHUK), same but with “29.VI.1952”; **North Korea.** 2♂ (TARI), Husen-Valley, Kankyo-Nando, 14.VII.1937, leg. Y. Yano; 5♂, 2♀ (TARI), Mt. Myoko (= Mt. Myohyang, 妙香山), 25.VII.1937, leg. M. Yamada; **South Korea**. 1♀ (TARI), Suigen, Keiki-Do, 3.VI.1933, leg. D. Okamoto; **Russia Far East**. 1♀ (MNHUK), Primorskii krai Lazovski Zapovehik, 3–14.V.2001, leg. M. Quest.

###### Redescription.

**Figure 22.**
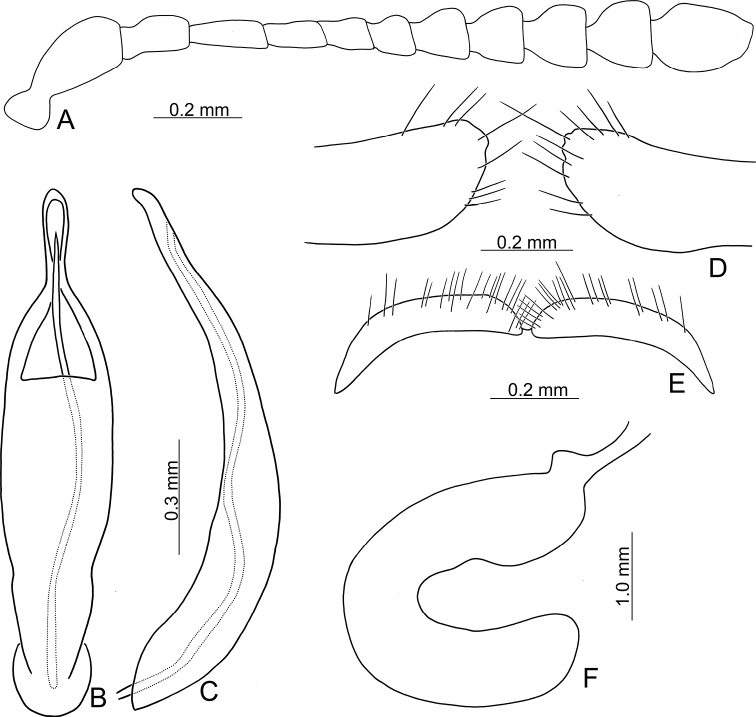
Diagnostic characters of Gonioctena (Brachyphytodecta) fulva (Motschulsky) **A** antenna **B** aedeagus, dorsal view **C** aedeagus, lateral view **D** gonocoxae **E** abdominal ventrite VIII **F** spermatheca.

Length 4.3–5.0 mm, width 2.8–3.5 mm. Body color (Fig. 8D–F) black; antennomeres I–IV, pronotum and elytra yellowish brown. Antennae (Fig. 22A) with antennomere III–V slender, VI slightly swollen, VII and VIII moderately swollen, IX and X strongly swollen, XI elongate oval, length ratios of antennomeres I–XI 1.0: 0.5: 0.5: 0.4: 0.4: 0.3: 0.4: 0.4: 0.5: 0.5: 0.7, length to width ratios of antennomeres I–XI 2.5: 1.9: 2.8: 2.2: 1.6: 1.0: 1.1: 1.0: 1.0: 1.0: 1.5. Pronotum 2.2–2.3× wider than long, lateral margins widest at base, convergent anteriorly, anterior angles strongly produced. Anterior and lateral margins bordered, lateral margins barely visible in dorsal view. Trichobothria absent on both anterior and posterior angles. Disc covered with sparse fine punctures and mixed with finer punctures; both sides covered with much larger, denser punctures. Scutellum distinctly wider than long, narrowed posteriorly. Elytra 1.2× longer than wide; lateral margins slightly wider posteriorly, widest near middle; humeral calli well developed; disc covered with regular coarse punctures arranged into single stria; interspaces covered with fine, sparse punctures. Hind wing well developed. Aedeagus (Fig. 22B, C) with apical process slender but short in dorsal view, 0.2× as long as aedeagus; lateral margins slightly narrowed in basal 1/3; moderately curved in lateral view; endophallic sclerite extremely elongate. Gonocoxae (Fig. 22D) wide, apical margin irregular, with several long setae along apical margins. Ventrite VIII (Fig. 22E) transverse, with several long setae along outer margin. Receptacle of spermatheca (Fig. 22F) slightly swollen, not separated from pump; pump long and curved; sclerotized proximal spermathecal duct moderately short.

###### Diagnosis.

Adults of Gonioctena (Brachyphytodecta) fulva are similar to yellowish brown adults of G. (B.) scutellaris but differ in possessing a black head, thoracic and abdominal ventrites (Fig. 8E) (yellowish brown head, thoracic and abdominal ventrites in G. (B.) scutellaris (Fig. 8B)) and short apical process of aedeagus (Fig. 22B) (extremely elongate apical process of aedeagus in G. (B.) scutellaris (Fig. 10B)).

###### Distribution.

China, Korea, Russia.

## ﻿Discussion

Our molecular phylogenetic analysis indicates that *G.tredecimmmaculata* is a monophyletic group, including Chinese and Taiwanese specimens. Molecular evidence supports *G.tredecimmmaculata* as an independent species with geographical variations in morphology.

Among Taiwanese species, only G. (Asiphytodecta) subgeminata and G. (Brachyphytodecta) scutellaris share the same host plant. Although their species identities are well established, niche separation deserves further study. Gonioctena (B.) liui sp. nov. is similar to the other consubgeneric species, G. (B.) scutellaris, based on larval and adult morphology. However, niche separation is complete since they utilize different plants as food sources. Moreover, molecular phylogenetic analysis revealed an independent clade of *G.liui* sp. Nov. with unambiguous morphological identification. Interspecies and intraspecies genetic distance analyses indicated that *G.liui* sp. nov. has reached species level. Molecular data support *G.liui* sp. nov. as a new species.

In addition, Baselga (2007) indicated that female genitalia, including abdominal tergite VIII, ventrite VIII, and gonocoxae are diagnostic for species identities of the subgenus Spartoxena with spermatheca reduced. This study supported this assertion. Moreover, abdominal ventrite VIII and gonocoxae are useful for identification of various species of the genus occurring in the same areas.

## Supplementary Material

XML Treatment for Gonioctena (Asiphytodecta) subgeminata

XML Treatment for Gonioctena (Asiphytodecta) tredecimmaculata

XML Treatment for Gonioctena (Brachyphytodecta) scutellaris

XML Treatment for Gonioctena (Brachyphytodecta) liui

XML Treatment for Gonioctena (s. str.) kamikawai

XML Treatment for Gonioctena (Sinomela) nigroplagiata

XML Treatment for Gonioctena (Sinomela) osawai

XML Treatment for Gonioctena (Brachyphytodecta) fulva

## Supplementary materials

Supplementary material 1 Collection information, taxon ID, and accession number of COI gene for each chrysomeline beetle Data type excel file Explanation note Collection information, taxon ID, and accession number of COI gene for each chrysomeline beetle. https://binary.pensoft.net/file/738742This dataset is made available under the Open Database License (http://opendatacommons.org/licenses/odbl/1.0/). The Open Database License (ODbL) is a license agreement intended to allow users to freely share, modify, and use this Dataset while maintaining this same freedom for others, provided that the original source and author(s) are credited.Chi-Feng Lee
